# Continuous High Frequency Deep Brain Stimulation of the Rat Anterior Insula Attenuates the Relapse Post Withdrawal and Strengthens the Extinction of Morphine Seeking

**DOI:** 10.3389/fpsyt.2020.577155

**Published:** 2020-10-14

**Authors:** Haigang Chang, Caibin Gao, Kuisheng Sun, Lifei Xiao, Xinxiao Li, Shucai Jiang, Changliang Zhu, Tao Sun, Zhe Jin, Feng Wang

**Affiliations:** ^1^Department of Neurosurgery, General Hospital of Ningxia Medical University, Yinchuan, China; ^2^Department of Neurosurgery, The First Affiliated Hospital of Xinxiang Medical University, Weihui, China; ^3^Ningxia Key Laboratory of Cerebrocranial Disease, Incubation Base of National Key Laboratory, Ningxia Medical University, Yinchuan, China; ^4^Department of Medical Cell Biology, Uppsala University, Uppsala, Sweden

**Keywords:** conditioned place preference, deep brain stimulation, substance use disorder, insula cortex, morphine dependence, proteomics

## Abstract

Deep brain stimulation (DBS) modulates the neuronal activity in specific brain circuits and has been recently considered as a promising intervention for refractory addiction. The insula cortex is the hub of interoception and is known to be involved in different aspects of substance use disorder. In the present study, we investigate the effects of continuous high frequency DBS in the anterior insula (AI) on drug-seeking behaviors and examined the molecular mechanisms of DBS action in morphine-addicted rats. Sprague-Dawley rats were trained to the morphine-conditioned place preference (CPP, day 1–8) followed by bilaterally implanted with DBS electrodes in the AI (Day 10) and recovery (Day 10–15). Continuous high-frequency (HF) -DBS (130 Hz, 150 μA, 90 μs) was applied during withdrawal (Day 16–30) or extinction sessions. CPP tests were conducted on days 16, 30, 40 during withdrawal session and several rats were used for proteomic analysis on day 30. Following the complete extinction, morphine-CPP was reinstated by a priming dose of morphine infusion (2 mg/kg). The open field and novel objective recognition tests were also performed to evaluate the DBS side effect on the locomotion and recognition memory. Continuous HF-DBS in the AI attenuated the expression of morphine-CPP post-withdrawal (Day 30), but morphine addictive behavior relapsed 10 days after the cessation of DBS (Day 40). Continuous HF-DBS reduced the period to full extinction of morphine-CPP and blocked morphine priming-induced recurrence of morphine addiction. HF-DBS in the AI had no obvious effect on the locomotor activity and novel objective recognition and did not cause anxiety-like behavior. In addition, our proteomic analysis identified eight morphine-regulated proteins in the AI and their expression levels were reversely changed by HF-DBS. Continuous HF-DBS in the bilateral anterior insula prevents the relapse of morphine place preference after withdrawal, facilitates its extinction, blocks the reinstatement induced by morphine priming and reverses the expression of morphine-regulated proteins. Our findings suggest that manipulation of insular activity by DBS could be a potential intervention to treat substance use disorder, although future research is warranted.

## Introduction

Substance use disorder is a chronic and relapsing brain disorder. It is characterized by compulsive drug-seeking and drug intake despite severe negative consequences, loss of control in limiting intake, and emergence of a negative emotional state (e.g., dysphoria, anxiety, irritability) when access to drug use is blocked (1). Substance use disorder causes enormous emotional, economic, medical, and legal costs to individuals and society and a global public health concern with high morbidity and mortality (2). Current treatments for substance use disorder including pharmacological and/or psychological interventions are available (3), but the relapse rates are still extremely high up to 50–70% (4). Additional approaches are needed to reduce relapse rate and strengthen efficacy of current strategies.

Deep brain stimulation (DBS) is a reversible, adjustable, minimally invasive, and safe neurosurgical intervention in which implanted electrodes deliver electrical pulses into certain targeted areas of the brain stereotactically. It has been widely used in the treatment of neurodegenerative diseases such as Parkinson's disease (5), dystonia, and tremor (6, 7) and in psychiatry for treatment resistant depression (8). The application of DBS to treat substance use disorder from both preclinical and clinical studies have showed a reduction in drug-seeking through stimulating (9).

The insula cortex plays a central role in the brain interoceptive system (10, 11) and is important in the neurocircuitry of addiction (1). Several studies have demonstrated that stroke-induced insula damage could lead to an abrupt and profound disruption of addiction to cigarette smoking without relapsing and craving (12, 13), and patients in the insula lesion group quit heroin use entirely at a higher rate than controls (14). In addition, an array of recent studies using animal models and pharmacological or chemical interventions have showed that insula is involved in different aspects of addictive behavior of various addictive drugs (15–20). For example, morphine-induced conditioned place preference (CPP) in rats were attenuated in studies using administration of muscarinic acetylcholine receptor antagonists and nitric oxide inhibitors into insula (21, 22). Previous work has also confirmed that insula is involved in the neurocircuitry underlying all stages of substance use disorder, and clearly suggested that insula is a fundamental brain region in the maintenance and relapse to addictive drugs (23).

Considering the involvement of insula in substance use disorder and the encouraging DBS results from preclinical and clinical studies, here, we examined the effects of continuous DBS in the anterior insula on the relapse of morphine addictive behavior post withdrawal, as well as extinction and priming-induced reinstatement of morphine seeking. Furthermore, we applied isobaric tags for relative and absolute quantitation labeling (iTRAQ)-based proteomic analysis in order to identify the proteins in the anterior insula regulated by DBS intervention of morphine addiction.

## Materials and Methods

### Animals

Male Sprague-Dawley rats weighting 230–270 g were used in this study. The rats were housed in animal rooms maintained under 12-h light/dark cycle (lights on at 08:00 AM to 08:00 PM) at 23−25°C and with moderate food restriction and tap water ad libitum and the weight of experimental animals was controlled between 280–300 g. Experimental rats were allowed to adapt to the laboratory environment for 1 week before behavioral experiments. Behavioral tests were performed at semidarkness condition during the light phase of the cycle. All procedures were in accordance with the Institutional Animal Care Use Committee of Ningxia Medical University, and approved by the Animal Ethics Committee of Ningxia Medical University.

### Drugs

Morphine (CAS number 21535-47-7, Shenyang First Pharmaceutical Factory, Shenyang, China) was administered at a dose of 10 mg/kg (s.c) showing drug reinforcement, which can induce conditioned place preference (CPP) without affecting movement (24).

### Conditioned Place Preference (CPP)

CPP apparatus is a plexiglass box composed of three chambers. Two side chambers of equal size (30 × 30 × 45 cm) were separated by a neutral chamber (5 × 10 × 45 cm) containing a removeable guillotine door. One side chamber had white walls and a grid gray floor, while the other chamber consisted of black walls and a frosted gray floor. The neutral area had a smooth floor with gray walls. The removable doors were inserted to restrict the animals to their paired environment during the conditioning phase, and removed away to allow the rats free access to both side chambers for testing. All CPP protocols were conducted at semi-darkness conditions. Behavioral data was acquired using video-tracking system (video behavior analysis software Smart 3.0, Panlab Company, Spain).

#### CPP Procedures

Animals were habituated to experimenter's handling and the experimental environment for 1 week. CPP experiment consisted of three phases: preconditioning, conditioning, post-conditioning. The “biased” procedure was used in this experiment (25, 26). In preconditioning phase, animals were allowed to explore freely between side chambers of CPP for 15 min and the rats that initially preferred the black chamber (55–75% of total spent time) were selected and progressed to the conditioning phase. Non-preferred side (white chamber, 25–45% of total spent time) was considered as baseline side preference. Conditioning phase consisted of eight training days with one conditioning trial each day for a total of eight trials. Rats were injected with morphine (morning on days 2, 6; afternoon on days 4, 8, respectively) and confined to the non-preferred side (white chamber; drug-paired side) for 45 min, and given saline (afternoon on days 1, 5; morning on days 3, 7) and restricted to the preferred side (black chamber; saline-paired side) for 45 min. During the conditioning phase, rats received a total of four injections of either morphine or saline on alternating days. Animals in the saline group were injected saline in two chambers, and the non-preferred side (white chamber) was used as reference baseline. In the post-conditioning phase, animals were conducted drug-free CPP tests, in which they were allowed free access to all CPP chambers (Figures 2A, 3A). The morphine side preference considered as index of conditioning was calculated and expressed as the percent of time spent in the morphine-paired chamber (white chamber) to the total time spent in both morphine-(white chamber) and saline-paired (black chamber) chambers.

#### Extinction Procedure

After stable acquisition of morphine-CPP and DBS electrode implantation, animals were placed in CPP chambers and allowed to move freely for 15 min, with no drug available. The procedure was conducted daily, until rats reached full extinction. The complete extinction was defined as the decline of the mean preference for morphine-paired chamber to the baseline value.

#### Reinstatement Procedure

One day after the complete extinction, rats were injected a priming-dose of morphine (2 mg/kg) in the CPP apparatus and immediately subjected to CPP tests.

### Experimental Groups and Design

Experimental rats for measuring morphine preference were allocated to four different groups: (1) the saline group (*n* = 15 for experiment 1; *n* = 12 for experiment 2), (2) morphine group that received alternate saline and morphine, without deep brain stimulation(DBS) apparatus implantation (*n* = 15 for experiment 1; *n* = 12 for experiment 2), (3) the morphine-DBS-sham group that received alternate saline and morphine, with DBS apparatus implantation but no active stimulation in all experiment procedures (*n* = 11 for experiment 1; *n* = 9 for experiment 2), (4) the morphine-DBS group that received alternate saline and morphine, with DBS apparatus implantation and continuous electrical stimulation in every experiment phase (*n* = 13 for experiment 1; *n* = 11 for experiment 2). Groups (1–4) rats were used in withdrawal relapse and extinction tests. The control drug-free rats for measuring the non-specific physiological effects of DBS were diveded into three group: (5) the control group (*n* = 6) without DBS apparatus implantation; (6) the DBS-sham group (*n* = 6) that with DBS apparatus implantation but no active electrical stimulation; (7) the DBS group (*n* = 6) that with DBS apparatus implantation and active electrical stimulation.

Experiment 1: The effect of continuous high frequency DBS (HF-DBS) of anterior insula (AI) on the relapse of morphine seeking behavior post withdrawal (Figure 2A).

The rats were trained to morphine conditioned place preference and then bilaterally implanted into the insula with DBS electrodes and allowed to recovery from surgery for 5 days. The rats underwent drug abstinence for 25 days. CPP tests were conducted on days 16, 30, and 40 during the drug withdrawal phase. In the morphine-DBS group, HF-DBS (130 Hz, 150 μA, 90 μs, rectangular stimulation pulse waveform) was continuously applied for 14 consecutive days (day 16–30). The morphine-DBS-sham rats received no active stimulation during the whole withdrawal session. The saline and morphine groups as controls received no surgery or stimulation. Total locomotion was analyzed during the preconditioning, post-conditioning and withdrawal phases by quantifying total distance traveled using Smart 3.0 video tracking system.

Experiment 2: The effect of continuous HF-DBS of AI on extinction and morphine-induced reinstatement of morphine seeking behavior (Figure 3A).

After acquisition of morphine-CPP and implanted with DBS apparatus, the rats underwent 10 days of extinction sessions and 1 day reinstatement test. HF-DBS (130 Hz, 150 μA, 90 μs, rectangular stimulation pulse waveform) was continuously applied for 11 days during extinction (Day 16–25) and reinstatement phase (Day 26) in morphine-DBS group but morphine-DBS-sham rats received no active stimulation during the whole extinction and reinstatement period.

**Figure 1:**
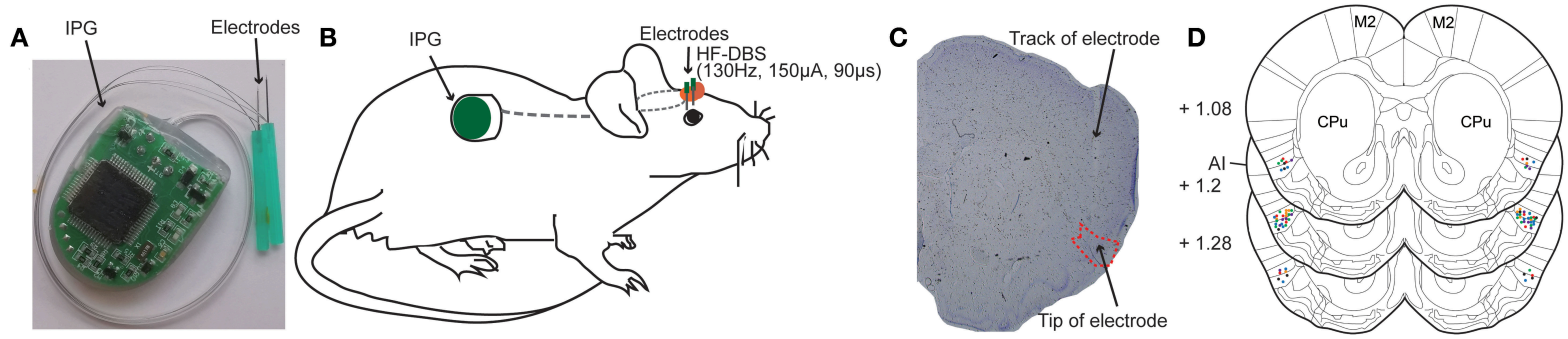
Deep brain stimulation (DBS) device and bilateral electrodes implantation in rat anterior insula (AI). **(A)** Photograph of the DBS device comprising an implantable pulse generator (IPG) and two electrodes. **(B)** Schematic view illustrating the electrodes that are secured in the skull over AI and the subcutaneously implanted IPG. **(C)** A representative rat brain section stained with cresyl violet showing the successfully placed electrode in the AI. **(D)** Locations of implanted electrodes (colored dots) in the AI of rats included for further analysis (*n* = 56). Experiment 1: morphine-DBS-sham (red), *n* = 11; morphine-DBS (green), *n* = 13. Experiment 2: morphine-DBS-sham (orange), *n* = 9; morphine-DBS (blue), *n* = 11. Open field and novel object recognition tests: DBS-sham (black), *n* = 6; DBS (purple), *n* = 6. CPu, caudate putamen. The brain coronal sections are adapted from the atlas of Paxinos and Watson (27).

Different from the stimulation electrodes used in the previous animal experiment studies, the DBS apparatus used in present study was designed and based on the DBS treatment system applied in the clinic. The DBS apparatus (Beijing PINS Medical Co., Ltd. Beijing, China) consisted of implantable electrical stimulation system and external programmer. The electrical stimulation system included bipolar electrodes, lead-extension and implantable pulse generator (IPG) (Figure 1A). Briefly, the implantable electrode is coaxial and the structure of its tip was shown as Figure 1A. The outer stainless-steel tube (diameter 0.3 mm) served as the reference pole. Its inner stainless-steel core (diameter 0.2 mm) was coated with an insulating Parylene layer and the uncoated tip served as the stimulating pole of negative polarity. The total length of the electrode is designed to be about 12 mm. The IPG was composed of microprocessor-controlled circuit board, button-type battery and electrode connection ports. The lead-extension was a flexible insulated coated wire, which connected with the electrodes end and the electrode connection ports of the pulse generator device. The total weight of the implantable DBS stimulation system was 5–7 g.

The external programmer communicated with the IPG through radio frequency. When the DBS stimulator was working *in vivo*, pulse parameters (pulse frequency, pulse width, pulse amplitude) and simulation mode (continuous or cyclic) can be set by the external programmer, and its current working state information such as its output parameters, battery capacity, and electrode impedance were displayed. The effective telemetry distance was 0–5 cm.

The rats were anesthetized by isoflurane inhalation (3–5% induction, 2–3% maintenance) and mounted in a stereotaxic apparatus. The body temperature was kept with the heating device. The IPG of DBS device was implanted in the back, and the electrodes and electrode-extension were threaded over neck and exited dorsally from the head subcutaneously (Figure 1B). Four stainless steel screws were fastened to the exposed skull. Corresponding to the target area of bilateral anterior insula, two holes with a diameter of 2 mm were drilled on the skull to expose to the dura mater. The electrodes were implanted into the anterior insula according to the following coordinates (27), relative to bregma: +1.2 mm anteroposterior(A/P), ±4.9 mm mediolateral(M/L), and −7.0 mm dorsoventral(D/V). Electrodes were cemented in place by affixing dental acrylic to the screws tightened into the skull and the excess plastic end of the electrodes were removed. One day after the surgical implantation, the rats recovered completely to the pre-operative state and maintained hygiene (grooming behavior). Throughout the experimental period, DBS functioned normally, and the rats were in good condition without any impact on their walking.

### Open Field Test

The open field test was used to measure locomotor activity and anxiety-like behavior in the current study. Eighteen rats were divided into control (*n* = 6), DBS-sham (*n* = 6) and DBS (*n* = 6) groups. One hour before testing, the rats were acclimatized to the testing room. Then, the rat was placed onto the central zone of the open field box facing one of the walls and allowed to explore for 10 min. Their exploring behavior was filmed using the video-tracking system and the data was analyzed by Smart 3.0 software package. The entire area of the open field box was cleaned with 70% ethanol and paper towel, before proceeding to the next rat. Continuous HF-DBS was applied 24 h prior to and during the behavior testing. Total traveled distance and time spent in the periphery (within 20 cm of the walls) of the arena were measured (28).

### Novel Object Recognition Test

After the open field test, the same rats then underwent the novel object recognition (NOR) test to evaluate the effect of DBS system on learning and memory (29). The experimental procedure consisted of three phases (habituation, training, testing) and was carried out in the same open field box, where the rats had been habituated to (day 7) when they were measured for locomotion and anxiety-like behavior. Twenty-four hours after the habituation (day 8), two identical subjects were presented 10 cm away from walls in opposite corners and each rat was allowed to explore the arena and objects for 10 min in the training phase. In the testing phase (day 9), one subject used during the training phase (i.e., the familiar subject) and one novel object were placed in the arena. The locations of the familiar and novel object were same as used during training phase for each rat. The rats were placed back into the arena and allowed to investigate the objects for 10 min. Exploration was defined as when the nose pointed at the object at a maximum distance of 2 cm from it and/or sniffing or touching it with snout. Time spent exploring the familiar and novel object during testing phase were recorded. The results were expressed as recognition index and calculated as the time spent exploring the novel object divided by the total time for the familiar object and novel object during the testing. Continuous HF-DBS was applied for all sessions in the DBS group.

### Verification of Electrode Position in Histology

At the conclusion of all experiments, the rats with implanted electrodes were given an overdose of pentobarbital (100 mg/kg) and transcardially perfused with saline followed by 4% paraformaldehyde. The brains were removed and immersed in 4% paraformaldehyde for 24 h, and then washed with phosphate-buffered saline (PBS) and submerged in 30% sucrose in PBS for 48 h. The brains were then frozen on dry ice and sliced (40 μm thick sections) using a cryostat microtome, and serial coronal sections were collected at the level of the insula. Sections were mounted on glass slides (coated with 2% gelatin), stained with Cresyl violet, covered with neutral balsam mounting media, and subsequently examined under a microscope (Leica, Germany) to verify the placement of the electrode.

### Real Time Quantitative PCR (RT-qPCR) Analysis

Three rats were bilaterally implanted with DBS electrodes in the anterior insula (AI) and received continuous electrical stimulation (130 Hz, 150 μA, 90 μs) for 14 days. RT-qPCR was performed to analyze the expression of activity-dependent genes (*Arc, c-fos*, and *Npas4*) in the AI of control and DBS rats. Total RNA was extracted from AI using TRIzol reagent (Invitrogen; Thermo Fisher Scientific, Inc.) and then reverse transcribed to cDNA using the PrimeScript™ RT Reagent Kit with gDNA Eraser (Takara) according to the manufacture's recommendations. All qPCR amplifications were performed in triplicates by a SYBR-based assay using SYBR Green PCR Master mix (Takara Biotechnology Co., Ltd.) and a 7,300 Real-Time PCR system (Applied Biosystems; Thermo Fisher Scientific, Inc.). The PCR reactions were performed with the following conditions: 2 min at 95°C, 40 cycles of 95°C for 15 s, 55°C for 30 s, 72°C for 30 s. Ubiquitin C (*Ubc*) served as the reference gene. The primer sequences (5′-3′) for *Arc, c-fos, Npas4* and *Ubc* are listed as follows: *Arc*, forward- AGTCTTGGGCAGCATAGCTC, reverse-GTATGAATCACTGCTGGGGGC; *c-fos*, forward- CCGACTCCTTCTCCAGCAT, reverse-TCACCGTGGGGATAAAGTTG; *Npas4*, forward- CTGCATCTACACTCGCAAGG, reverse- GCCACAATGTCTTCAAGCTCT; *Ubc*, forward- ACACCAAGAAGGTCAAACAGGA, reverse-CACCTCCCCATCAAACCCAA. The relative changes in gene expression were calculated using 2^−ΔCq^ method and Cq is the quantification cycle.

### iTRAQ-Based Proteomics Analysis

#### Tissue Preparation, Protein Extraction and Quantification

The animals (three rats from each saline, morphine, and morphine-DBS groups in experiment 1) were anesthetized by inhalation of isoflurane (3–5%) and decapitated immediately after the behavioral test on day 30 during withdrawal phase. The whole brain was removed and placed into the brain slice mold on ice and 1-mm thick slices close to the electrode channel were obtained. The anterior insula was isolated and lysed in the lysis buffer (100 mM ammonium bicarbonate, 8 M urea, 0.2% SDS, pH = 8). The extracted protein pellets were dissolved in the buffer (6 M urea, 100 mM triethylammonium bicarbonate TEAB, pH = 8.5) and the protein concentration was determined by Bradford protein quantitative kit.

#### iTRAQ Labeling of Peptides and LC-MS/MS Analysis

The protein samples were trypsinized in TEAB buffer overnight, mixed with formic acid and centrifuged for 5 min at room temperature. The supernatant was loaded to the C18 desalting column and eluted by elution buffer (0.1% formic acid, 70% acetonitrile). The eluents were collected, lyophilized, reconstituted with TEAB buffer and mixed with iTRAQ labeling reagent with shaking for 2 h at room temperature. The reaction was stopped by adding 50 mM Tris-HCl (pH = 8). All labeling samples were fractionated in a Rigol L-3000 HPLC system. Retained peptides were eluted with iTRAQ Mobile phase A (2% acetonitrile, pH = 10) and B (98% acetonitrile, pH = 10). Ten fractions were collected and lyophilized under vacuum. For transition library construction, shotgun proteomics analyses were performed using an EASY-nLCTM 1200 UHPLC system (Thermo Fisher) coupled with an Q Exactive HF(X)mass spectrometer (Thermo Fisher) operating in the data-dependent acquisition (DDA) mode. Sample was injected into a C18 Nano-Trap column (2 cm × 75 μm, 3 μm). Peptides were separated in an analytical column (15 cm × 150 μm, 1.9 μm), using a linear gradient elution. The separated peptides were analyzed by Q Exactive HF(X)mass spectrometer (Thermo Fisher). The top 40 precursors of the highest abundant in the full scan were selected and fragmented by higher energy collisional dissociation (HCD) and analyzed in MS/MS. The raw data of MS detection was named as “.raw.”

#### Protein Identification and Quantification

The resulting spectra from each run were searched separately according to protein database by the search engines: Proteome Discoverer 2.2 (PD 2.2, Thermo). The searched parameters are set as follows: mass tolerance for precursor ion was 10 ppm and mass tolerance for product ion was 0.02 Da. Carbamidomethyl was specified as fixed modifications, Oxidation of methionine (M) and iTRAQ plex were specified as dynamic modification, acetylation and iTRAQ plex were specified as N-Terminal modification in PD 2.2. A maximum of two miscleavage sites were allowed.

In order to improve the quality of analysis results, the software PD 2.2 further filtered the retrieval results: Peptide Spectrum Matches (PSMs) with a credibility of more than 99% was identified PSMs. The identified protein contains at least one unique peptide. The identified PSMs and protein were retained and performed with FDR (false discovery rate) no more than 1.0%. The protein quantitation results were statistically analyzed by Student's *t*-test. Differentially expressed proteins (DEPs) were identified as proteins with a fold change ratio > 1.2 or <0.83 (*p* < 0.05).

### Statistical Analysis

Data are presented as mean ± standard error of the mean (SEM). Two-way repeated measures analysis of variance (ANOVA) were performed to determine statistical differences in morphine preference experiments. Bonferroni *post hoc* analyses were used for multiple comparisons. One-way ANOVA was used to assess differences in locomotion activity, anxiety-like behavior, and novel object recognition. Multiple unpaired Student's *t*-tests were used to analyze qPCR results. *P* < 0.05 was considered statistically significant. SPSS 23.0 (SPSS Inc., Chicago, IL, USA) and GraphPad Prism 8 (GraphPad Software, La Jolla, CA) were used to perform all statistical analyses and plot graphs, respectively.

## Results

### Verification of DBS Electrode Placement

The DBS device consists of an implantable pulse generator (IPG) and two electrodes (Figure 1A). We bilaterally implanted the electrodes to rat anterior insula (AI) and placed the IPG subcutaneously in the rat (Figure 1B). After the completion of all behavior tests, the animals were sacrificed followed by the removal of the electrodes. We further prepared brain coronal sections stained with cresol violet and verified the placement of electrodes (Figures 1C,D). Only the rats with correct electrode placement in the AI were included in the analysis of behavior tests. The locations of electrode tips in the AI from different groups of animals were displayed in Figure 1D.

### Continuous High Frequency-DBS of Anterior Insula Suppresses Relapse of Morphine-Induced Conditioned Place Preference (CPP) During the Withdrawal Stage

**Figure 2:**
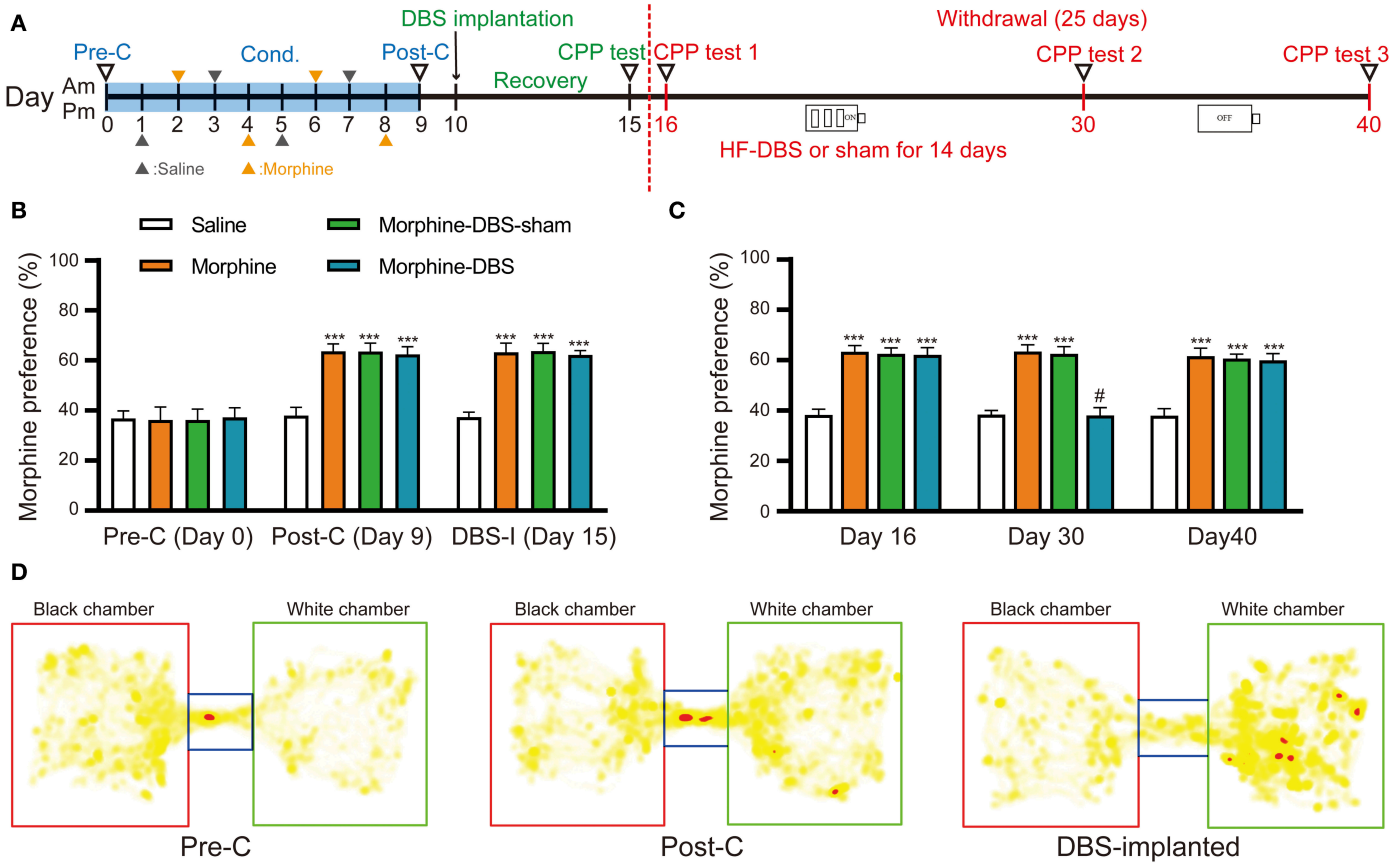
Continuous high frequency-DBS (HF-DBS) of anterior insular (AI) suppresses morphine-induced conditioned place preference (CPP). **(A)** Experimental timeline. The morphine CPP protocol starts with a pre-conditioning test (Day 0, pre-C) followed an 8-day conditioning (i.p. 10 mg/kg morphine or 0.9% saline on alternating days) and a post-conditioning test (Day 9, post-C). Once the morphine CPP was successfully established, the rats were bilaterally implanted with DBS electrodes to AI (Day 10) followed by a 5-day recovery and a CPP test was performed (Day 15, DBS-I). The rats further underwent morphine abstinence for 25 days. HF-DBS (130 Hz, 150 μA, 90 μs) was continuously applied for the first 14 days (Day 16–30) and CPP tests were performed (Day 16, 30, and 40). **(B)** The 8-day morphine conditioning induced significantly higher morphine preferences than the saline control group (Bonferroni's *post hoc* test, day 9, post-C, ***p < 0.001 vs. saline control) [phase: *F*_(4, 47)_ = 332.771, *p* < 0.001; group: *F*_(3, 50)_ = 323.751, *p* < 0.001; interaction: *F*_(12, 147)_ = 20.674, *p* < 0.001]. Morphine elicited significant side preference in electrodes-implanted rats and the morphine-induced CPP persisted 5-day after the implantation of DBS electrodes (Bonferroni's *post hoc* test, day 15, ***p < 0.001 vs. saline control). **(C)** Continuous HF-DBS (Day 16–30) reduced morphine-induced CPP on day 30 as compared with the morphine-DBS sham group (Bonferroni's *post hoc* test, day 30, ***p < 0.001 vs. saline group, #*p* < 0.001 vs. morphine- and morphine-sham-DBS). The morphine-induced CPP recurred 10 days after turning off DBS in the morphine-DBS group (Day 40) (***p < 0.001 vs. saline group). **(D)** Representative heat maps of CPP tests in electrodes-implanted before morphine conditioning and post electrodes implantation. White chamber: morphine-paired chamber; black chamber: saline-paired chamber. Data are shown as mean with SEM. Two-way repeated ANOVA with *post-hoc* Bonferroni test, *n* = 15 for saline and morphine, *n* = 11 for morphine- DBS -sham and *n* = 13 for morphine-DBS groups.

To evaluate whether our HF-DBS protocol is sufficient to modulate the activity in AI, we analyzed the expression of activity-dependent genes (*Arc, c-fos*, and *Npas4*) in AI of rats that received continuous HF-DBS (130 Hz, 150 μA, 90 μs) for 14 days. The levels of *c-fos* and *Npas4* but not *Arc* transcripts were significantly increased in DBS rats compared to control rats (Supplementary Figure 1). The injection of morphine (10 mg/kg) for eight alternate days resulted in a significant preference for morphine-paired white chamber in three groups of rats (morphine, morphine-DBS-sham, and morphine-DBS), as compared with the saline-treated group (Figures 2A,B, day 9, post-C, two-way repeated measures-ANOVA followed by Bonferroni multiple comparison test, *p* < 0.001) [phase: *F*_(4, 47)_ = 332.771, *p* < 0.001; group: *F*_(3, 50)_ = 323.751, *p* < 0.001; interaction: *F*_(12, 147)_ = 20.674, *p* < 0.001]. We further implanted bilateral DBS electrodes in two groups of rats (morphine-DBS-sham, and morphine-DBS) on day 10 followed by recovery for 5 days (Figure 2A). On day 15, the expression of morphine CPP was persistent in both morphine group and electrode-implanted groups (morphine-DBS-sham and morphine-DBS, Figures 2A,B,D). All groups of rats underwent subsequent 25-day period of abstinence for drugs and the CPP tests were performed on day 16, 30, and 40 (Figure 2A). For morphine-DBS rats, the IPG delivering continuous HF-DBS was turned on for 14 consecutive days (Day 16–30) followed by 10-day off (Day 31–40). On day 30, the morphine CPP was significantly reduced in DBS rats, as compared with both morphine and morphine-DBS-sham rats (Figure 2C, Bonferroni *Post hoc* comparisons analysis, *p* < 0.001). In contrast, Bonferroni's test showed that there was no significant among morphine-DBS (61.9 and 59.9%), morphine-DBS-sham (62.5 and 60.6%), and morphine (63.3 and 61.6%) groups in the expression of morphine-CPP on days 16 (*p* = 0.615, *p* = 0.222, *p* = 0.462) or 40 (*p* = 0.558, *p* = 0.154, *p* = 0.558) (Figure 2C). Therefore, continuous HF-DBS suppressed the morphine CPP during the withdrawal stage. We also analyzed the total distance traveled during CPP tests in saline, morphine, morphine-DBS-sham, and morphine-DBS groups. Two-way ANOVA showed that there was no significant change in total distance traveled among the four groups during the preconditioning, post-conditioning and withdrawal phases [phase: *F*_(4, 164)_ = 0.401, *p* = 0.808; group: *F*_(3, 41)_ = 0.075, *p* = 0.973; interaction: *F*_(12, 164)_ = 1.091, *p* = 0.371].

### Continuous HF-DBS Accelerates Extinction and Prevents Priming-Induced Relapse of Morphine-Induced CPP

**Figure 3:**
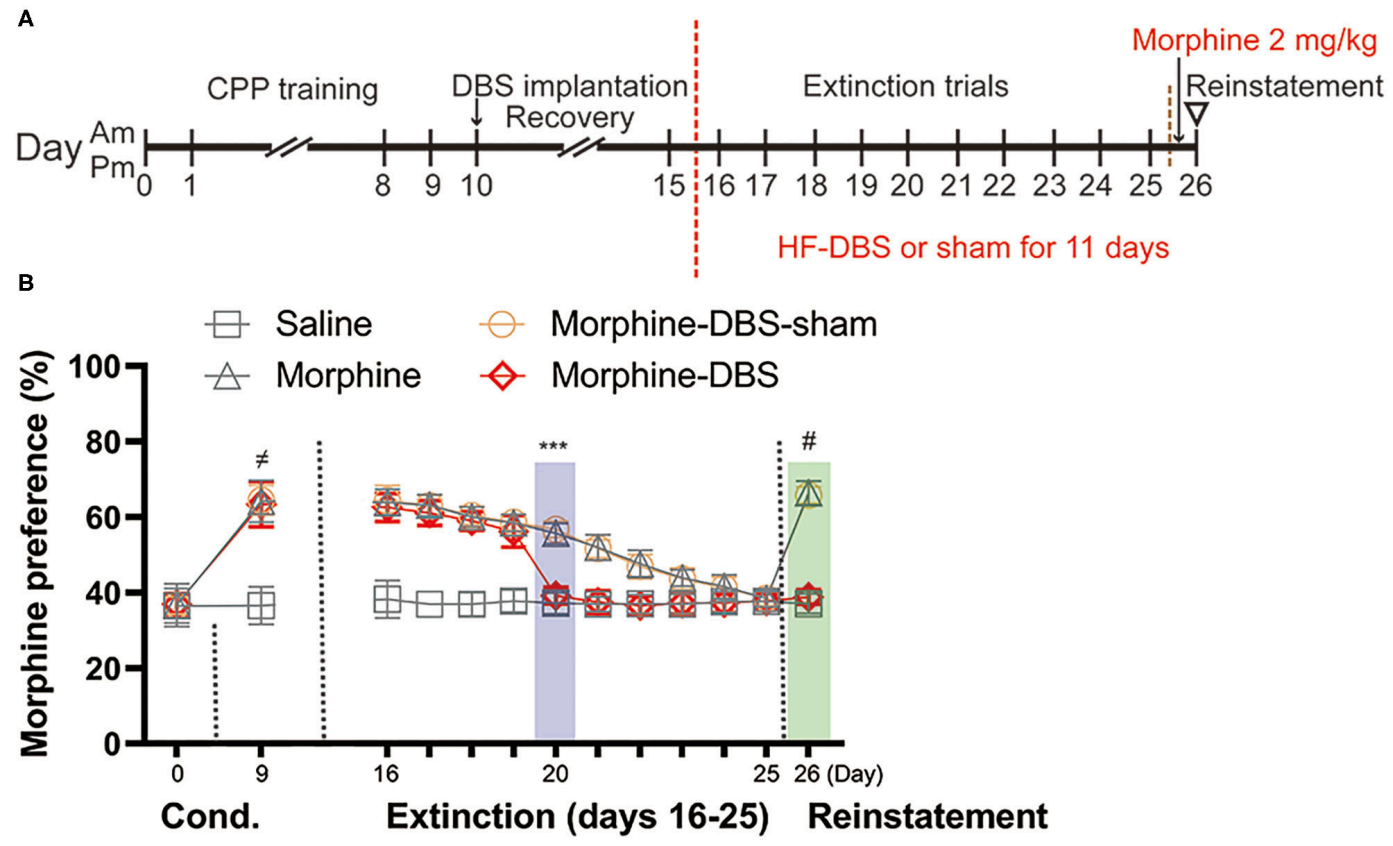
Continuous high frequency-DBS (HF-DBS) of anterior insula (AI) accelerates extinction and prevents priming-induced relapse of morphine-induced conditioned place preference (CPP). **(A)** Experimental scheme. After an 8-day morphine-CPP training, the rats were bilaterally implanted with DBS electrodes to AI (morphine-DBS and morphine-DBS-sham groups) followed by a 5-day recovery. All groups of rats were further subjected to drug-free daily extinction sessions (Day 16–25). HF-DBS (130 Hz, 150 μA, 90 μs) was continuously applied in morphine-DBS rats. Twenty-four hours after the complete extinction, all groups of rats received a priming dose of morphine (2 mg/kg) followed by a CPP test. **(B)** The morphine-DBS group expressed the complete extinction of morphine-induced CPP on day 20, which were 5 days earlier than both morphine and morphine-DBS-sham groups (Two-way repeated-measures ANOVA, phase: *F*_(12, 29)_ = 195.486, *p* < 0.001; treatment: *F*_(3, 40)_ = 152.651, *p* < 0.001; interaction: *F*_(36, 93)_ = 5.392, *p* < 0.001; *Post hoc* test, Extinction day 5: *p* < 0.001 for morphine-DBS vs. morphine-DBS-sham and morphine). On day 26, the priming injection of morphine failed to reinstate morphine-induced CPP in the morphine-DBS group (*p* < 0.001 for morphine or morphine-DBS-sham vs. saline or morphine-DBS). Data are shown as mean with SEM. Two-way repeated ANOVA with *post hoc* Bonferroni test. ^≠^*p* < 0.001, Saline vs. morphine or morphine-DBS or morphine-DBS-sham. ***p < 0.001, morphine-DBS vs. morphine or morphine-DBS-sham. ^#^*p* < 0.001, morphine or morphine-DBS-sham vs. saline or morphine-DBS. *n* = 12 for saline and morphine, *n* = 9 for morphine- DBS -sham and *n* = 11 for morphine-DBS groups.

Next, we evaluated whether continuous HF-DBS could affect extinction and prevent subsequent recurrence of morphine-CPP. Rats from morphine-DBS group as well as morphine-DBS-sham, morphine and saline groups received 10 days of extinction trials (Day 16–25, drug-free CPP tests) followed by a morphine reinstatement on day 26 (Figure 3A). Continuous HF-DBS (130 Hz, 150 μA, 90 μs) was delivered to AI of morphine-DBS rats during this period, whereas morphine-DBS-sham rats receive no electrical stimulation. In morphine-DBS rats, 5-day extinction trains completely eliminated the expression of morphine CPP on day 20, which was significantly different from morphine and morphine-DBS-sham rats (Figures 3A,B, Two-way repeated-measures ANOVA, [phase: *F*_(12, 29)_ = 195.486, *p* < 0.001; treatment: *F*_(3, 40)_ = 152.651, *p* < 0.001; interaction: *F*_(36, 93)_ = 5.392, *p* < 0.001]; *Post hoc* test, Extinction day 20: *p* < 0.001 for morphine-DBS vs. morphine-DBS-sham and morphine). In contrast, 10-day extinction trials resulted in a complete extinction in morphine and morphine-DBS-sham rats on day 25 (Figure 3B). These findings suggest continuous HF-DBS of AI facilitates extinction of morphine place preference.

Twenty-four hours after the complete extinction of morphine place preference, each rat was given a priming dose injection of morphine (2 mg/kg) and CPP test was immediately conducted for assessing associative reward/drug-associated memory. On day 26, morphine and morphine-sham-DBS rats showed significant reinstatement of preference to the morphine-paired side as compared with their previous test on day 25 (38.4% → 66.4% for morphine, *p* < 0.001; 38.4% → 65.8% for morphine-sham-DBS, *p* < 0.001, Figure 3B) and as compared with morphine-DBS or saline rats (*p* < 0.001, Figure 3B). The above results indicate continuous HF-DBS prevents extinguished morphine-seeking relapse induced by a priming dose injection.

### Continuous HF-DBS of Anterior Insula (AI) Does Not Influence Locomotor Activity, Anxiety-Like Behavior and Novel Object Recognition

**Figure 4:**
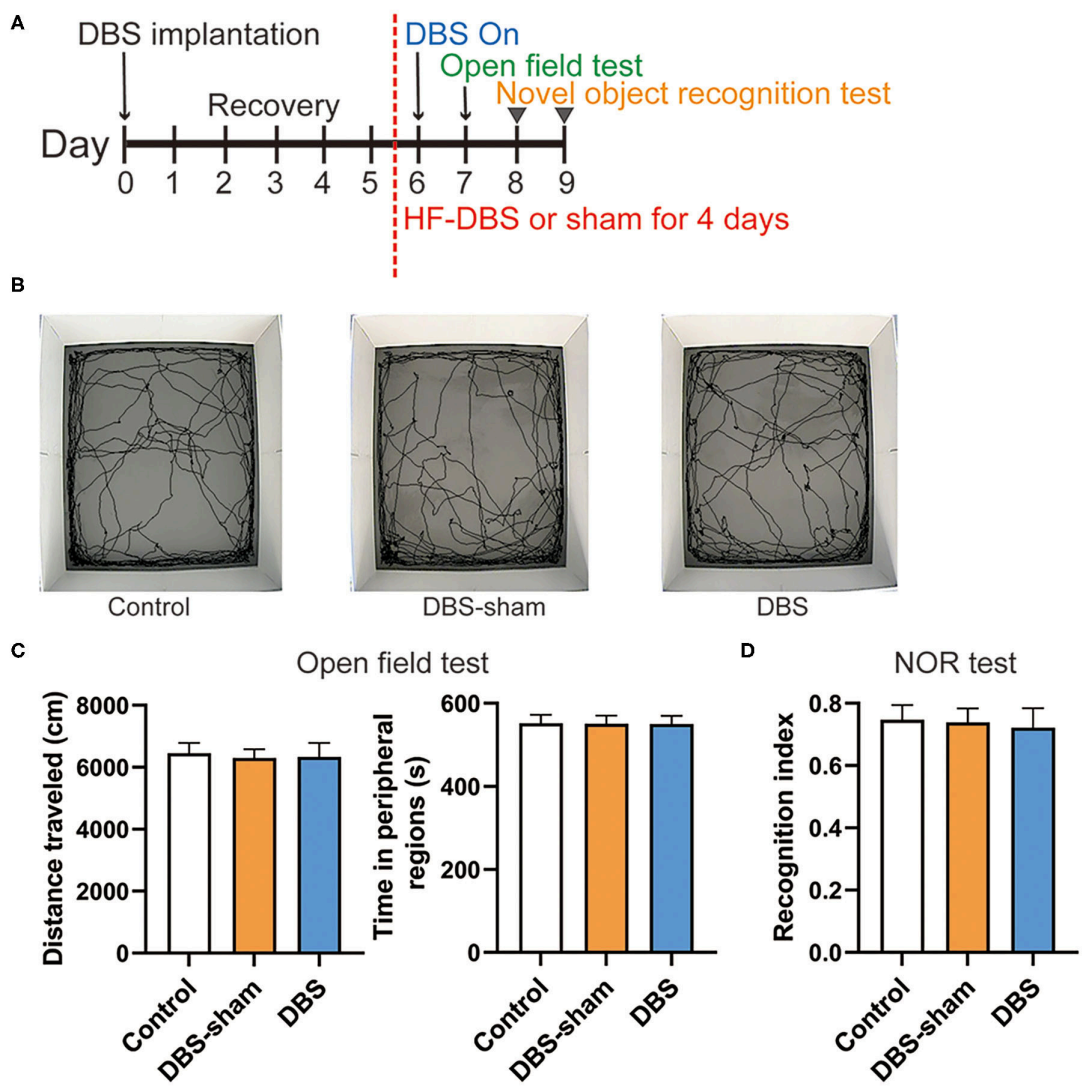
Behavior performance of rats after the continuous high frequency DBS (HF-DBS) stimulus of anterior insula (AI). **(A)** Experimental scheme. The rats in DBS and DBS-sham groups were bilaterally implanted with DBS electrodes to AI followed by a 5-day recovery. HF-DBS (130 Hz, 150 μA, 90 μs) was continuously applied in DBS-rats between day 6 and 9. The open field test was performed on day 7 followed by the novel object recognition (NOR) test on day 8 and 9. **(B)** Representative track maps of rats from control, DBS-sham and DBS groups in the open field tests. **(C)** Total traveled distance in the arenas and time spent in the peripheral regions were measured in the open field tests to assess the locomotor activity and anxiety-like behavior, respectively. There were no significant differences among groups on locomotor activity [*F*_(2, 17)_ = 0.307, *p* = 0.740] and in the time spent in the peripheral zones of the open-field arena [*F*_(2, 17)_ = 0.022, *p* = 0.978]. **(D)** The NOR test was performed to evaluate memory and cognitive functions. The recognition index was calculated by the ratio of time spent exploring the novel object to the total time of both novel and familiar objects. There were no significant differences in the recognition index among control, DBS-sham and DBS groups [*F*_(2, 17)_ = 0.355, *p* = 0.707]. Data are shown as mean with SEM. *n* = 6 for three groups.

We further examined whether continuous HF-DBS caused non-specific effects. The locomotor activity and anxiety-like behavior were evaluated by the open field test. After 5-day recovery from electrode implantation, continuous HF-DBS (130 Hz, 150 μA, 90 μs) was delivered to AI in DBS rats for 24 h prior to and during the test task (Figure 4A). The DBS-sham rats received no active electrical stimulation. One-way ANOVA showed that there were no significant differences in the total traveled distance in the open field area [*F*_(2, 17)_ = 0.307, *p* = 0.740] and the time spent in the peripheral zones of the open-field [*F*_(2, 17)_ = 0.022, *p* = 0.978] among control, DBS and DBS-sham rats (Figures 4B,C).

The NOR test was subsequently performed to assess the effect of DBS on recognition memory. HF-DBS was continuously applied to AI in DBS rats during the habituation, familiarization and choice phases of the test (Figure 4A). The recognition index did not differ among the control, DBS-sham and DBS groups [Figure 4D, one-way ANOVA, [*F*_(2, 17)_ = 0.355, *p* = 0.707]], suggesting continuous HF-DBS of AI does not impair recognition memory.

### Identification of Differential Expression Proteins in the AI Associated With HF-DBS Intervention of Morphine Addiction by iTRAQ-Based Proteomics Analysis

**Figure 5:**
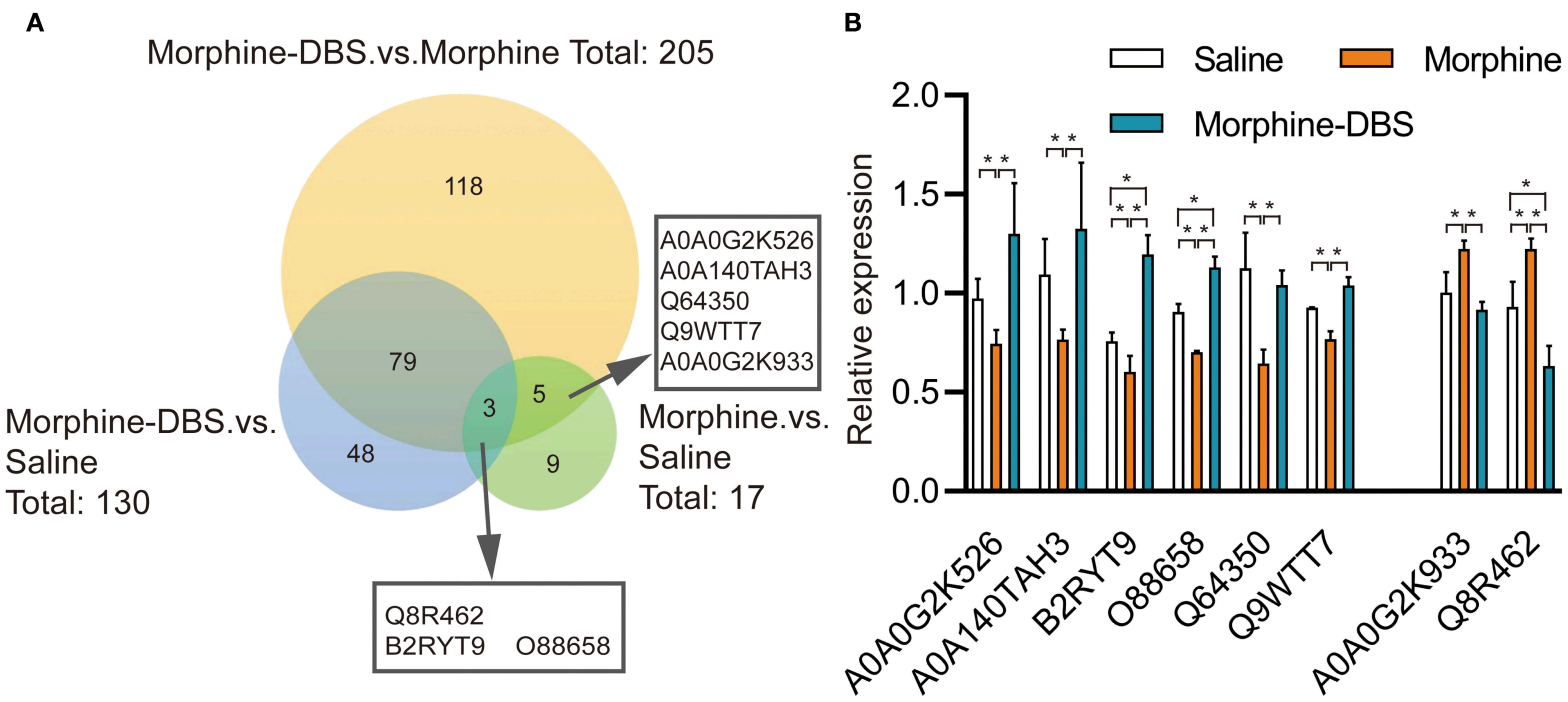
HF-DBS reverses the expression of morphine addiction-related proteins in the anterior insula during the withdrawal phase. **(A)** Venn diagram for the number of differentially expressed proteins (DEPs) identified by the three comparisons morphine-DBS vs. morphine, morphine vs. saline and morphine-DBS vs. saline. Eight DEPs were identified commonly in the two comparisons (morphine-DBS vs. morphine and morphine vs. saline) and 3 out of 8 DEPs were also identified in the morphine-DBS vs. saline comparison. **(B)** Relative expressions of 8 differential expressed proteins among saline, morphine, and morphine-DBS groups. Six proteins (A0A0G2K526, B2RYT9, A0A140TAH3, O88658, Q64350, and Q9WTT7) were significantly down-regulated in the morphine group as compared with the saline group (morphine vs. saline, *p* < 0.05) but up-regulated in the morphine-DBS (morphine-DBS vs. morphine, *p* < 0.05), whereas two proteins (A0A0G2K933 and Q8R462) increased in the morphine group (morphine vs. saline, *p* < 0.05) but decreased in the morphine-DBS (morphine-DBS vs. morphine, *p* < 0.05). The expression of differentially expressed protein was assessed with unpaired Student's *t*-tests. *p < 0.05. *n* = 3 for saline, morphine, and morphine-DBS groups.

We performed the quantitative proteomic approach based on iTRAQ coupled with 2D-LC MS/MS to identify the key proteins in the AI associated with DBS therapy of morphine addiction. We prepared AI protein samples from three animals in each saline, morphine, and morphine-DBS groups in experiment 1 immediately after the behavioral test on day 30 during withdrawal phase. A total of 4,650 non-redundant proteins were identified by global proteomic analysis with >99% confidence in correct sequence identification. Proteins with significant quantitative difference among groups (ratio fold change > 1.2 or <0.83, *p* < 0.05) were defined as differential expression proteins (DEPs). The list of DEPs identified by the comparisons (morphine-DBS vs. morphine, morphine vs. saline, morphine-DBS vs. saline) is shown in Supplementary Table 1. Our results showed that eight DEPs were commonly present in the comparison groups of morphine-DBS vs. morphine and morphine vs. saline and 3 out of 8 DEPs (Q8R462, B2RYT9, and O88658) were also identified in the morphine-DBS vs. saline comparison (Figure 5A). These eight common DEPs were A0A0G2K526 (Guanine nucleotide-binding protein G[olf] subunit alpha), A0A0G2K933 (Eukaryotic translation initiation factor 4E family member 2), B2RYT9 (Translational activator of cytochrome c oxidase 1), A0A140TAH3 (Glutamate-rich WD repeat-containing protein 1), O88658 (Kinesin-like protein KIF1B), Q64350(Translation initiation factor eIF-2B subunit epsilon), Q8R462 (Amino acid transporter), Q9WTT7 (Basic leucine zipper and W2 domain-containing protein 2) (Table 1). Notably, six proteins (A0A0G2K526, B2RYT9, A0A140TAH3, O88658, Q64350, and Q9WTT7) were significantly deceased in the morphine group post withdrawal as compared with saline group, but increased in the morphine-DBS group (Figure 5B and Table 1). In contrast, the expression levels of two proteins (A0A0G2K933 and Q8R462) were significantly down-regulated in the morphine group but up-regulated after DBS intervention (Figure 5B and Table 1).

**Table 1:** The list of differentially expressed proteins in the anterior insula that overlap between morphine vs. saline and morphine-DBS vs. morphine and morphine-DBS vs. saline comparison.

**Protein ID**	**Protein name**	**Morphine vs. Saline**			**Morphine-DBS vs. Morphine**			**Morphine-DBS vs. Saline**		
		**Down/Up**	**Fold Change**	***P*-value**	**Down/Up**	**Fold Change**	***P*-value**	**Down/Up**	**Fold Change**	***P*-value**
A0A0G2K526	Guanine nucleotide-binding protein G(olf) subunit alpha	Down	0.7648	0.0294	Up	1.7456	0.0217			
A0A0G2K933	Eukaryotic translation initiation factor 4E family member 2	Up	1.2197	0.0267	Down	0.7490	0.0007			
B2RYT9	Translational activator of cytochrome c oxidase 1	Down	0.7964	0.0443	Up	1.9842	0.0012	Up	1.5803	0.0020
A0A140TAH3	Glutamate-rich WD repeat-containing protein 1	Down	0.7002	0.0391	Up	1.7305	0.0461			
O88658	Kinesin-like protein KIF1B	Down	0.7763	0.0125	Up	1.6088	0.0001	Up	1.2489	0.0049
Q64350	Translation initiation factor eIF-2B subunit epsilon	Down	0.5732	0.0127	Up	1.6132	0.0026			
Q8R462	Amino acid transporter	Up	1.3149	0.0213	Down	0.5170	0.0008	Down	0.6797	0.0338
Q9WTT7	Basic leucine zipper and W2 domain-containing protein 2	Down	0.8280	0.0028	Up	1.3536	0.0015		

## Discussion

Here, we provide direct experimental evidence that DBS of anterior insula is a potential approach to treat substance use disorder. Using a rodent morphine addiction model, we have demonstrated continuous HF-DBS of anterior insula suppresses the morphine-induced CPP during the withdrawal stage, facilitates the extinction and prevents the priming-induced relapse of morphine CPP. We also have shown that continuous HF-DBS of anterior insula did not alter locomotor activity during open field and CPP tests, anxiety-like behavior and novel object recognition in rats.

In this study, we first established a stable morphine CPP rat model, followed by DBS electrode implantation and stimulation intervention, mimicking the clinical treatment situation in pre-clinical models (30, 31). In addition, we used a new-designed implantable stimulus generator which was programmed through the skin by an external wireless device. This new DBS apparatus allows unrestricted moving and feeding of experimental animals and delivers long-term chronic stimulation (at the effective voltage for over 3 months), which is more advantageous than the majority of previous DBS devices used for rodent models (32, 33). We have observed that HF-DBS increases the expression of activity-dependent genes *c-fos* and *Npas4* in the AI, which suggests our stimulation protocol modulates AI cellular activity and is in line with previous DBS studies (34–37).

The insula cortex is considered as the central hub of interoception that plays an important role in the onset and maintenance of substance use disorder (38–42). Pharmacological inactivation or lesion of the anterior/posterior insula can reduce relapse of drug seeking (15, 16, 18, 19, 43). For instance, a reversible inactivation of the anterior insula by injection of muscimol and baclofen decreased relapse to methamphetamine seeking after voluntary abstinence in a rat model (44). Moreover, HF-DBS of the rat insular region significantly attenuated nicotine-taking, under both schedules of reinforcement, as well as nicotine-seeking behavior induced by cues and priming (17). Our finding that the relapse of morphine CPP and morphine priming-induced reinstatement are blocked by continuous HF-DBS is in line with aforementioned studies, suggesting the anterior insula is a potential neuromodulation target in the prevention of drug relapse. The high-frequency electrical stimulation has been shown to inhibit neuronal activity of the stimulated area and cause a functional lesion, although the electrical effects of DBS are strongly influenced by the parameters (single pulse or continuous stimulation, amplitude, voltage, polarity, frequency, pulse width, pulse shape) and temporal aspects of stimulation itself (45). Consistent with our findings, previous studies have shown that HF-DBS in various brain regions could affect different aspects of drug reinforcement. For example, HF-DBS of the orbitofrontal cortex significantly blocked the establishment and reinstatement of morphine induced CPP (46). HF-DBS of the bilateral nucleus accumbens prevented the morphine-induced reinstatement of morphine seeking in the CPP test and accelerated the rate of decay of drug craving in morphine-preference rats (47). Interestingly, HF-DBS of the dorsal but not ventral region of the ventral striatum impaired extinction in morphine-CPP (35).

The establishment of drug-chamber association is a process of experiencing and evaluating unprecedented drug use interoceptive effects. During the withdrawal and extinction phase, when animals are placed in CPP chambers, interceptive central representative of drug use in insula is reactivated, which promotes associative reward/drug-associated memory through interoception processing (48, 49). Therefore, in this study animals tend to stay in morphine-associated context for a longer time in order to obtain the expected interoceptive effect of drug use. This is a form of decision-making behavior that is influenced by drug use interoception processing. Because the anterior insula is involved in the process of interoception processing and decision-making in associative reward/drug-associated memory, continuous HF-DBS of this area disturbs the preference for morphine reward.

The insular cortex is a part of the cerebral cortex, and connects with addiction-related areas to form a wide range of addictive brain network, and plays an important role in the three stages of substance use disorder cycle (23). Through its projections to the pre-frontal cortex, the amygdala and the ventral striatal nodes of the corticostriatal circuitry, the anterior insula influences executive functions and reward-related behavior (50). In rodents, the anterior insula is associated with decision-making (51), impulsivity, and vulnerability to develop compulsive behavior (50). The damage or inactivation of anterior insula can also affect the intake of nicotine, cocaine seeking and the response to cocaine related cues (19, 52), suggesting a role of insular mediated interoceptive mechanism in the reinforcement effect and incentive properties of addictive drugs, which can easily increase the desire for addictive drugs and guide reward seeking behavior (53, 54). HF-DBS inactivated neurons in the insular cortex of stimulated area and significantly decreased nicotine self-administration and cue- and priming-induced reinstatement of nicotine seeking. These neurons required longer durations of HF-stimulation to elicit this response (17). Similarly, the results of this study also showed that the relapse of morphine CPP occurred again on day 40 post withdrawal after 10 days of cessation of DBS. Therefore, continuous HF-DBS inactivated neurons in the stimulated area, prevent the further processing of the information of drug use interoceptive effect and output the signal to the downstream target involved in the high-level cognitive decision-making process.

This study has explored the effect of HF-DBS in insular on morphine addiction animal models, but whether LF-DBS or the combination of HF and LF-DBS can also affect the development of morphine addictive behaviors requires further studies. In addition, the therapeutic effect of DBS depends on different stimulation parameters that have not been examined in detail in this study. Previous studies have shown that administration of HF-DBS in different brain areas either in extinction (45) or reinstatement sessions (55, 56) could prevent the reinstatement of drug seeking during the reinstatement session. However, in this study we applied HF-DBS to AI in both extinction and reinstatement sessions and could not distinguish the long-term or acute therapeutic effect of DBS. Substance use disorder is a chronic recurrent brain disorder, so the exact therapeutic effect of DBS remains to be determined.

Our proteomic analysis reveals that 17 proteins are differentially expressed in the anterior insula after 8-day morphine exposure and subsequent withdrawal as compared with saline-treated rats. Previous studies have reported that the expression levels of four DEPs (CaM kinase-like vesicle-associated protein, Guanine nucleotide-binding protein G[olf] subunit alpha, Serine protease inhibitor A3K, Striated muscle-specific serine/threonine-protein kinase) are altered in the brain or heart by morphine treatment and withdrawal (57–59). The rest of 13 DEPs are not identified in the Morphinome Database (addiction-proteomics.org) which includes the morphine-regulated proteins from 29 published proteomics studies (60). The discrepancy can be explained by a variety of factors, for example, morphine doses and times of exposure, types of animals, tissues and brain sub-regions. Another important finding of this study is that the expression of 8 out of 17 DEPs are reversed by HF-DBS intervention. For example, guanine nucleotide-binding protein (olf) subunit alpha (Gα_olf_) is down-regulated in the anterior insula of morphine-treated rats but up-regulated in DBS-morphine rats. Gα_olf_ is a subunit of trimeric GTP-binding protein (G-protein) stimulating adenylyl cyclase enzymes, which regulate the cAMP signaling pathway and further modulate brain functions such as synaptic plasticity drug dependence (59, 61). In the rat brain, Gα_olf_ is mainly expressed in olfactory neurons and striatum (62), but it is also present in other sub-regions including the pre-frontal cortex (63). In agreement with our results, a previous study has shown that chronic morphine treatment decreases the expression of Gα_olf_ in the striatal presynaptic fractions of rats (57). Similarly, repeated injection of amphetamine or cocaine results in a markedly decrease of Gα_olf_ in the rat nucleus accumbens (64, 65). Although the role of altered Gα_olf_ expression in drug dependence remains unclarified, the decreased expression of Gα_olf_ may reduce the coupling of G-protein-coupled receptor (GPCR)-Gα_olf_ -adenylyl cyclase and downstream signaling pathways (66). The effects of DBS on the cellular and molecular level are complex (45), but our findings that DBS reverses the expression of several morphine-regulated proteins may provide some clues to the underlying mechanisms of DBS suppression of morphine-induced CPP.

One limitation of this study is that only male animals were used. Although more than 80% of the addiction research is conducted in males, accumulating evidence has showed that addiction occurs differently in males and females (67–71). Compared to males, females are more likely to make a rapid transition from casual drug use to dependence, experience higher levels of craving and relapse during withdrawal, and consume more drugs during relapse (72, 73). During abstinence, females experience stronger drug cravings (74). This is mainly due to the ovarian hormone cycle in females, and short-term exposure to estradiol will increase drug availability (75). Animal studies further indicate that estradiol may be necessary for the development of substance use disorder. In rodents, short-term estradiol intake in female rats enhanced the acquisition and escalation of drug intake, drug abuse motivation and relapse like behavior (76–78). These findings suggest that sex and hormonal status are the main determinants of substance use disorder, and it is essential to include both males and females in addiction studies. The effects of HF-DBS in female morphine-addiction rats will be further explored.

In conclusion, we have shown that continuous HF-DBS in the bilateral anterior insula prevents the relapse of morphine place preference after withdrawal, facilitates its extinction, blocks the reinstatement induced by morphine priming and reverse the expression of morphine-regulated proteins, but does not affect locomotor activity, anxiety-like behavior and novel object recognition. In view of previous work and this study, manipulation of insular activity by pharmacological or other means, such as DBS, can be a potential intervention to treat substance use disorder.

## Data Availability Statement

The original contributions presented in the study are publicly available. This data can be found at ProteomeXchange Consortium (http://proteomecentral.proteomexchange.org) via the iProX partner repository (79) with the dataset identifier PXD021122.

## Ethics Statement

The animal study was reviewed and approved by Animal Ethics Committee of Ningxia Medical University.

## Author Contributions

ZJ and FW participated in the study design, data collection, analysis of data, and preparation of the manuscript. HC, CG, and KS carried out the experimental work and the data collection and interpretation and wrote the first draft. LX, XL, SJ, CZ, and TS carried out the data collection and interpretation. All authors read and approved the final manuscript.

## Conflict of Interest

The authors declare that the research was conducted in the absence of any commercial or financial relationships that could be construed as a potential conflict of interest.
