# Supplementary material for: Continuous High Frequency Deep Brain Stimulation of the Rat Anterior Insula Attenuates the Relapse Post Withdrawal and Strengthens the Extinction of Morphine Seeking
Source: Front Psychiatry. 2020 Oct 14;11:577155. doi: 10.3389/fpsyt.2020.577155 (PMC7591677; doi:10.3389/fpsyt.2020.577155)
Supplement: Supplementary file 1 [file Data_Sheet_1.doc]

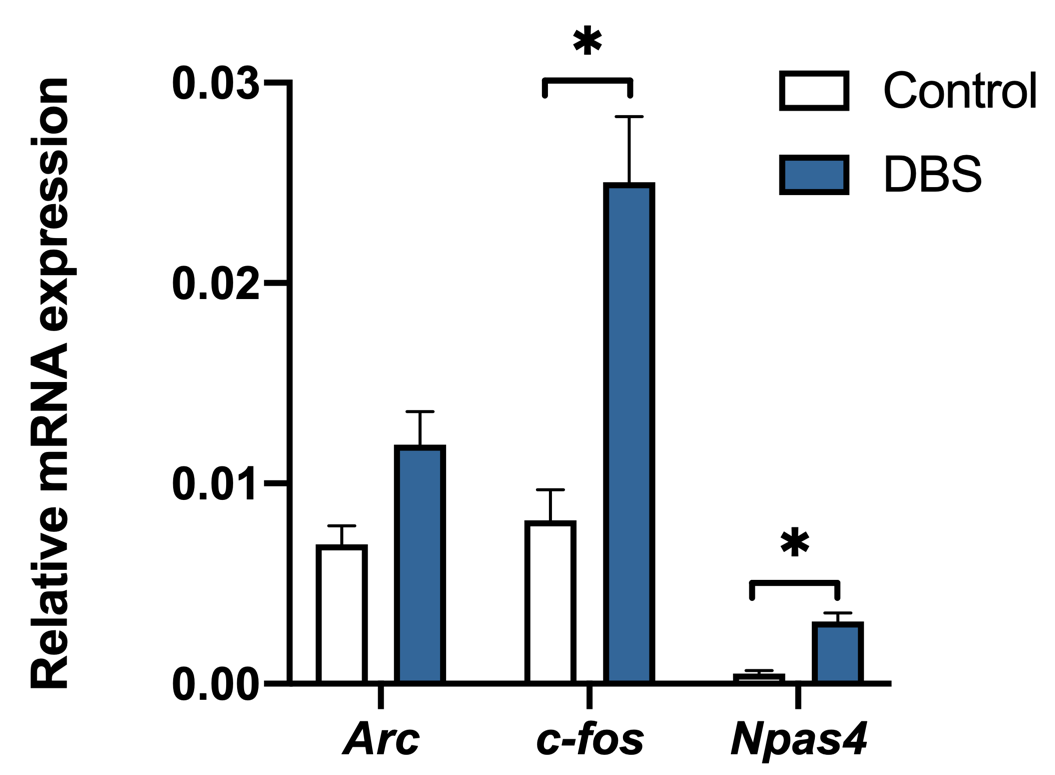


**Supplementary Fig 1.** Comparisons of *Arc*, *c-fos* and *Npas4* mRNA expression levels in the AI of rats with HF-DBS for 14 days and control using RT-qPCR. The expression levels of *c-fos* and *Npas4* were significant higher in the DBS rats than controls (DBS vs control; *c-fos*, adjusted p value = 0.019; *Npas4*, adjusted p value = 0.014, 3 animals in each group).

**Supplementary Table 1. The list of differentially expressed proteins (DEPs) in the anterior insular cortex from rats in saline, morphine and morphine-DBS groups.**

| **Protein ID** | **Protein names** | **Gene names** | **P-value** | **Log_2_ relative fold change (FC)** |
| --- | --- | --- | --- | --- |
| **Morphine vs. Saline** |  |  |  |  |
| A0A0G2K1R5 | CaM kinase-like vesicle-associated protein | *Camkv* | 0.0473 | -0.5200 |
| A0A0G2K526 | Guanine nucleotide-binding protein G(olf) subunit alpha | *Gnal* | 0.0294 | -0.3867 |
| A0A0G2K933 | Eukaryotic translation initiation factor 4E family member 2 | *Eif4e2* | 0.0267 | 0.2865 |
| A0A140TAH3 | Glutamate-rich WD repeat-containing protein 1 | *Grwd1* | 0.0391 | -0.5140 |
| B2RYT9 | Translational activator of cytochrome c oxidase 1 | *Taco1* | 0.0443 | -0.3283 |
| D3ZC46 | Transcription factor 25 | *Tcf25* | 0.0342 | 0.2999 |
| E7CXR8 | Receptor protein-tyrosine kinase | *Egfr* | 0.0166 | -0.2892 |
| M0RBQ1 | mRNA-decapping enzyme 2-like | *Dcp2* | 0.0140 | -0.3154 |
| M0RCY7 | UFM1-specific peptidase 1 | *Ufsp1* | 0.0442 | -0.2692 |
| O88658 | Kinesin-like protein KIF1B | *Kif1b* | 0.0125 | -0.3652 |
| P05545 | Serine protease inhibitor A3K | *Serpina3k* | 0.0397 | -0.9278 |
| P14141 | Carbonic anhydrase 3 | *Ca3* | 0.0163 | -0.6396 |
| Q63638 | Striated muscle-specific serine/threonine-protein kinase | *Speg* | 0.0138 | -0.4207 |
| Q64350 | Translation initiation factor eIF-2B subunit epsilon | *Eif2b5* | 0.0127 | -0.8027 |
| Q91XV6 | FXYD domain-containing ion transport regulator 6 | *Fxyd6* | 0.0034 | -0.3362 |
| Q9WTT7 | Basic leucine zipper and W2 domain-containing protein 2 | *Bzw2* | 0.0028 | -0.2722 |
| Q8R462 | Amino acid transporter (Fragment) |  | 0.0213 | 0.3950 |
| **Morphine-DBS vs. Morphine** |  |  |  |  |
| A0A0A0MXV3 | Cell growth regulator with EF hand domain 1, isoform CRA_b | *Cgref1* | 0.0013 | -0.3267 |
| A0A0A1FZ38 | NADH-ubiquinone oxidoreductase chain 3 | *ND3* | 0.0243 | 0.2721 |
| A0A0G2JSJ8 | Alpha-L-fucosidase | *Fuca1* | 0.0036 | 0.2648 |
| A0A0G2JSR6 | Guanine nucleotide-binding protein subunit gamma | *Gng7* | 0.0112 | 0.2828 |
| A0A0G2JTJ9 | RCG50655 | *Tfcp2* | 0.0420 | 0.3485 |
| A0A0G2JU15 | Pentatricopeptide repeat domain 3 | *Ptcd3* | 0.0347 | 0.3098 |
| A0A0G2JWG6 | Golgin B1 | *Golgb1* | 0.0064 | -0.4345 |
| A0A0G2JZ88 | Similar to hypothetical protein A530094D01 | *RGD1306556* | 0.0310 | -0.3642 |
| A0A0G2JZB7 | Neuron navigator 3 | *Nav3* | 0.0086 | -0.3901 |
| A0A0G2K0D3 | Leiomodin-1 | *Lmod1* | 0.0250 | -0.2646 |
| A0A0G2K121 | Unc-119 lipid-binding chaperone B | *Mlec* | 0.0010 | -0.3115 |
| A0A0G2K132 | Formin-like 2 | *Fmnl2* | 0.0361 | 0.2934 |
| A0A0G2K526 | Guanine nucleotide-binding protein G(olf) subunit alpha | *Gnal* | 0.0218 | 0.8038 |
| A0A0G2K5N8 | ArfGAP with coiled-coil, ankyrin repeat and PH domains 3 | *Acap3* | 0.0151 | -0.2991 |
| A0A0G2K6N9 | COMM domain containing 4 (Predicted), isoform CRA_c | *Commd4* | 0.0404 | 0.3844 |
| A0A0G2K7A6 | Transporter | *Slc6a7* | 0.0185 | 0.2953 |
| A0A0G2K933 | Eukaryotic translation initiation factor 4E family member 2 | *Eif4e2* | 0.0008 | -0.4168 |
| A0A0G2K9B1 | Serine protease inhibitor A3N | *LOC299282* | 0.0158 | 0.3372 |
| A0A0G2QC15 | HIV-1 Tat interactive protein 2 | *Htatip2* | 0.0011 | 0.5177 |
| A0A0H2UHB3 | Carbonic anhydrase 4 | *Ca4* | 0.0286 | -0.2703 |
| A0A0H2UHP1 | Retinal dehydrogenase 1 | *Aldh1a1* | 0.0051 | -0.4352 |
| A0A140TAA3 | Ral GTPase-activating protein subunit alpha-1 | *Ralgapa1* | 0.0213 | 0.3059 |
| A0A140TAA5 | WD repeat domain phosphoinositide-interacting protein 4 | *Wdr45* | 0.0325 | 0.3984 |
| A0A140TAD1 | ADP-sugar pyrophosphatase | *Nudt5* | 0.0064 | -0.3273 |
| A0A140TAH3 | Glutamate-rich WD repeat-containing protein 1 | *Grwd1* | 0.0462 | 0.7912 |
| A0A1W2Q678 | Uncharacterized protein | *FAM120C* | 0.0008 | 0.4090 |
| A0A452Q762 | BTB domain-containing 8 | *Btbd8* | 0.0178 | -0.3478 |
| A1L1I6 | Reticulon | *Rtn3* | 0.0240 | 0.5030 |
| A2RRU1 | Glycogen [starch] synthase, muscle | *Gys1* | 0.0182 | 0.2977 |
| B0BN15 | Similar to src homology 2 domain-containing transforming protein D | *Shd* | 0.0098 | -0.3681 |
| B0BN35 | LOC687694 protein | *Elp4* | 0.0160 | 0.4271 |
| B0BNA5 | Coactosin-like protein | *Cotl1* | 0.0000 | 0.2657 |
| B0BNE7 | Tetraspanin | *Tspan7* | 0.0041 | -0.3823 |
| B0K025 | Oligosaccharyltransferase complex subunit OSTC | *Ostc* | 0.0218 | 0.4645 |
| B2GUY4 | Dematin actin-binding protein | *Dmtn* | 0.0102 | -0.2684 |
| B2RYI8 | Papss1 protein | *Papss1* | 0.0006 | -0.2811 |
| B2RYQ8 | Large subunit ribosomal protein L36a, isoform CRA_a | *Rpl36a* | 0.0447 | -0.3206 |
| B2RYT9 | Translational activator of cytochrome c oxidase 1 | *Taco1* | 0.0012 | 0.9886 |
| B2RYW4 | Mitochondrial ribosomal protein L53 | *Mrpl53* | 0.0021 | -0.6191 |
| B2RYW8 | MICOS complex subunit Mic10 | *Micos10* | 0.0344 | -0.3107 |
| B2RZ61 | Carbonic anhydrase 7 | *Car7* | 0.0007 | -0.3088 |
| B2RZB5 | Charged multivesicular body protein 2A | *Chmp2a* | 0.0137 | -0.4084 |
| B3DMA0 | Tumor protein p53-inducible protein 11 | *Tp53i11* | 0.0286 | 0.3084 |
| B5DFM8 | BCAS2, pre-mRNA-processing factor | *Bcas2* | 0.0138 | 0.4393 |
| D0IN10 | SH3 and cysteine rich domain 2 (Predicted), isoform CRA_a | *Stac2* | 0.0395 | -0.2723 |
| D1M8S3 | Interleukin 1 receptor accessory protein b | *Il1rap* | 0.0272 | -0.3159 |
| D3Z949 | Similar to RIKEN cDNA 2900010M23 | *RGD1561671* | 0.0028 | 0.2968 |
| D3ZAF6 | ATP synthase subunit f, mitochondrial | *Atp5mf* | 0.0005 | 0.3481 |
| D3ZB51 | Protein turtle homolog B | *Igsf9b* | 0.0002 | 0.3255 |
| D3ZCL3 | U1 small nuclear ribonucleoprotein C | *Snrpc* | 0.0230 | -0.3117 |
| D3ZDR2 | Charged multivesicular body protein 6 | *Chmp6* | 0.0111 | -0.2800 |
| D3ZEI0 | Ribosomal_L28e domain-containing protein | *0.0307* | -0.3442 |  |
| D3ZH39 | Eph receptor B3 | *Ephb3* | 0.0048 | -0.3020 |
| D3ZHA0 | Filamin-C | *Flnc* | 0.0383 | 0.3181 |
| D3ZHV3 | Metallothionein | *Mt1m* | 0.0429 | -0.2959 |
| D3ZHV7 | Serine dehydratase-like | *Sdsl* | 0.0007 | 0.5785 |
| D3ZJK8 | PhoLip_ATPase_N domain-containing protein | *0.0049* | 0.2943 |  |
| D3ZJR6 | SMCR8-C9orf72 complex subunit | *Smcr8* | 0.0156 | 0.2839 |
| D3ZQN3 | PNMA family member 8B | *Pnma8b* | 0.0004 | 0.3483 |
| D3ZSL2 | ABRA C-terminal-like | *Abracl* | 0.0266 | 0.3342 |
| D3ZTK0 | Tetratricopeptide repeat domain 9B | *Ttc9b* | 0.0236 | -0.2774 |
| D3ZUM4 | Beta-galactosidase | *Glb1* | 0.0333 | 0.2768 |
| D3ZWS0 | Scribble planar cell polarity protein | *Scrib* | 0.0011 | 0.2790 |
| D3ZWU9 | Required for meiotic nuclear division 5 homolog A | *Rmnd5a* | 0.0138 | 0.5392 |
| D3ZZN4 | Uncharacterized protein | *0.0348* | -0.2814 |  |
| D4A1G1 | Acylphosphatase | *Acyp2* | 0.0060 | -0.2930 |
| D4A1H2 | Phosphatidylinositol-specific phospholipase C, X domain-containing 3 | *Plcxd3* | 0.0285 | 0.4395 |
| D4A206 | Treacle ribosome biogenesis factor 1 | *Tcof1* | 0.0319 | -0.5105 |
| D4A2K5 | Nuclear receptor interacting protein 3 (Predicted), isoform CRA_a | *Nrip3* | 0.0308 | -0.3931 |
| D4A3X6 | Phospholipid-transporting ATPase | *Atp8a2* | 0.0168 | -0.2851 |
| D4A626 | Calmin | *Clmn* | 0.0017 | 0.2837 |
| D4A6N8 | T-box brain transcription factor 1 | *Tbr1* | 0.0160 | -0.2902 |
| D4A7Q6 | Zinc finger protein 428 | *Zfp428* | 0.0286 | -0.3086 |
| D4A7U6 | U6 snRNA-associated Sm-like protein LSm3 | *Lsm3* | 0.0014 | 0.4166 |
| D4A9Q5 | Carboxypeptidase M | *Cpm* | 0.0003 | 0.6574 |
| D4AAU6 | Coiled-coil domain containing 25 (Predicted) | *Ccdc25* | 0.0050 | 0.2657 |
| D4ABY0 | Complexin 3 | *Cplx3* | 0.0098 | -0.3069 |
| D4ACK1 | Nucleoporin 214 (Fragment) | *Nup214* | 0.0110 | -0.3185 |
| D4AEL3 | Distal membrane arm assembly complex 1 | *Dmac1* | 0.0035 | -0.6590 |
| E9PT53 | Wolfram syndrome 1 homolog (Human) | *Wfs1* | 0.0026 | 0.3349 |
| E9PTK4 | Threonine synthase-like 1 | *Thnsl1* | 0.0491 | 0.3782 |
| F1LQ09 | Atlastin GTPase 2 | *Atl2* | 0.0181 | -0.3569 |
| F1LQI6 | Zyg-11-related, cell cycle regulator | *Zer1* | 0.0072 | 0.3204 |
| F1LQP9 | Transportin 1 | *Tnpo1* | 0.0325 | 0.2800 |
| F1LQS3 | 60S ribosomal protein L6 | *Rpl6-ps1* | 0.0250 | -0.3868 |
| F1LUC0 | Similar to RIKEN cDNA 5730410E15 gene (Predicted), isoform CRA_a | *Sybu* | 0.0010 | 0.4288 |
| F1LW74 | IQ motif-containing GTPase-activating protein 2 | *Iqgap2* | 0.0003 | 0.2775 |
| F1LZF2 | Formin-binding protein 1 | *Fnbp1* | 0.0046 | -0.2987 |
| F1M0N1 | Tyrosine-protein kinase | *Abl2* | 0.0096 | -0.3700 |
| F1M1B3 | WASH complex subunit 5 | *Washc5* | 0.0172 | 0.2831 |
| F1M1W4 | Nedd4 family-interacting protein 2 | *Ndfip2* | 0.0326 | -0.3258 |
| F1M3B1 | Ankyrin repeat domain-containing protein 29-like | *LOC108348154* | 0.0248 | -0.4645 |
| F1M3H8 | Hypothetical protein LOC681410 | *LOC681410* | 0.0019 | 0.5170 |
| F2W8A6 | Neuropeptide Y | *Npy* | 0.0144 | 0.5443 |
| F6Q5K7 | Mitochondrial ribosomal protein S18B | *Mrps18b* | 0.0070 | -0.4095 |
| F7FDM3 | Mitochondrial translational initiation factor 2 | *Mtif2* | 0.0467 | 0.2638 |
| G3V6M3 | Synaptotagmin II | *Syt2* | 0.0202 | -0.3165 |
| G3V6X7 | ProSAAS | *LOC108348172* | 0.0023 | 0.2717 |
| G3V6Z5 | Glutamate receptor 3 | *Gria3* | 0.0019 | -0.2960 |
| G3V7W1 | Programmed cell death protein 6 | *Pdcd6* | 0.0046 | 0.4091 |
| G3V8Y5 | DNA-directed RNA polymerase subunit beta | *Polr2b* | 0.0029 | 0.4321 |
| G3V913 | Heat shock 27kDa protein 1 | *Hspb1* | 0.0017 | 0.7674 |
| M0R3N4 | Vesicle amine transport 1-like | *Vat1l* | 0.0226 | 0.3881 |
| M0R4J7 | Dishevelled-binding antagonist of beta-catenin 3 | *Dact3* | 0.0171 | -0.2947 |
| M0R665 | 60S ribosomal protein L29 | *0.0116* | -0.4769 |  |
| M0R6T1 | TatD DNase domain-containing 1 | *Tatdn1* | 0.0467 | 0.3979 |
| M0R6V0 | Ubiquitin-like domain-containing protein | *0.0305* | -0.4240 |  |
| M0R715 | Patatin-like phospholipase domain-containing 6 | *Pnpla6* | 0.0225 | 0.4053 |
| M0RAD5 | ATP-dependent Clp protease proteolytic subunit | *Clpp* | 0.0224 | 0.3223 |
| M0RAT6 | Transmembrane p24 trafficking protein 8 | *Tmed8* | 0.0086 | 0.4914 |
| M0RBF1 | Complement C3 | *C3* | 0.0012 | 0.3224 |
| O35263 | Platelet-activating factor acetylhydrolase IB subunit gamma | *Pafah1b3* | 0.0016 | 0.3351 |
| O54701 | Sodium/potassium/calcium exchanger 2 | *Slc24a2* | 0.0329 | -0.4249 |
| O88279 | Slit homolog 1 protein | *Slit1* | 0.0073 | 0.4438 |
| O88658 | Kinesin-like protein KIF1B | *Kif1b* | 0.0002 | 0.6860 |
| P01048 | T-kininogen 1 | *Map1* | 0.0078 | 0.7648 |
| P02625 | Parvalbumin alpha | *Pvalb* | 0.0015 | -0.7248 |
| P06399 | Fibrinogen alpha chain | *Fga* | 0.0338 | 0.2858 |
| P06536 | Glucocorticoid receptor | *Nr3c1* | 0.0125 | 0.3998 |
| P07171 | Calbindin | *Calb1* | 0.0035 | -0.2838 |
| P08483 | Muscarinic acetylcholine receptor M3 | *Chrm3* | 0.0157 | -0.3289 |
| P14669 | Annexin A3 | *Anxa3* | 0.0408 | 0.2704 |
| P17078 | 60S ribosomal protein L35 | *Rpl35* | 0.0472 | -0.3159 |
| P20059 | Hemopexin | *Hpx* | 0.0232 | 0.3848 |
| P28492 | Glutaminase liver isoform, mitochondrial | *Gls2* | 0.0419 | 0.2707 |
| P30835 | ATP-dependent 6-phosphofructokinase, liver type | *Pfkl* | 0.0003 | 0.2855 |
| P31647 | Sodium- and chloride-dependent GABA transporter 3 | *Slc6a11* | 0.0133 | -0.2644 |
| P31652 | Sodium-dependent serotonin transporter | *Slc6a4* | 0.0379 | 0.2839 |
| P47196 | RAC-alpha serine/threonine-protein kinase | *Akt1* | 0.0023 | 0.4600 |
| P47728 | Calretinin | *Calb2* | 0.0363 | 0.2897 |
| P47819 | Glial fibrillary acidic protein | *Gfap* | 0.0084 | 0.6348 |
| P52944 | PDZ and LIM domain protein 1 | *Pdlim1* | 0.0362 | -0.2723 |
| P55051 | Fatty acid-binding protein, brain | *Fabp7* | 0.0236 | 0.3158 |
| P55063 | Heat shock 70 kDa protein 1-like | *Hspa1l* | 0.0024 | -0.5581 |
| P60756 | MAM domain-containing glycosylphosphatidylinositol anchor protein 2 | *Mdga2* | 0.0010 | -0.3208 |
| P62628 | Dynein light chain roadblock-type 1 | *Dynlrb1* | 0.0013 | 0.2787 |
| P62775 | Myotrophin | *Mtpn* | 0.0058 | -0.2712 |
| P63090 | Pleiotrophin | *Ptn* | 0.0335 | -0.2797 |
| P80299 | Bifunctional epoxide hydrolase 2 | *Ephx2* | 0.0083 | -0.2712 |
| P80432 | Cytochrome c oxidase subunit 7C, mitochondrial | *Cox7c* | 0.0035 | -0.3512 |
| Q01066 | Calcium/calmodulin-dependent 3,5-cyclic nucleotide phosphodiesterase 1B | *Pde1b* | 0.0345 | 0.3441 |
| Q02765 | Cathepsin S | *Ctss* | 0.0091 | 0.4294 |
| Q03626 | Murinoglobulin-1 | *Mug1* | 0.0024 | -0.3122 |
| Q0D2L6 | Ras-related GTP-binding C | *Rragc* | 0.0209 | 0.2947 |
| Q1RP74 | RCG53953, isoform CRA_a | *LOC103690005* | 0.0291 | -0.3086 |
| Q2MCP5 | WD repeat domain 45B | *Wdr45b* | 0.0198 | 0.4692 |
| Q32PX9 | AFG1-like ATPase | *Afg1l* | 0.0443 | 0.2740 |
| Q3KRE2 | Methyltransferase like 7A, isoform CRA_b | *Mettl7a* | 0.0001 | 0.6778 |
| Q3KRE3 | Guanine nucleotide-binding protein subunit gamma | *Gng10* | 0.0297 | 0.2650 |
| Q3MHU5 | Reticulophagy regulator 2 | *Retreg2* | 0.0203 | 0.3439 |
| Q3MIE4 | Synaptic vesicle membrane protein VAT-1 homolog | *Vat1* | 0.0239 | 0.2825 |
| Q3ZAU5 | DDHD domain-containing 1 | *Ddhd1* | 0.0197 | -0.3922 |
| Q4FZT8 | SPRY domain-containing protein 4 | *Spryd4* | 0.0030 | 0.2700 |
| Q4FZX5 | Methionine-R-sulfoxide reductase B2, mitochondrial | *Msrb2* | 0.0003 | -0.2807 |
| Q4KM45 | UPF0687 protein C20orf27 homolog | *0.0120* | 0.4467 |  |
| Q4KM73 | UMP-CMP kinase | *Cmpk1* | 0.0142 | 0.3066 |
| Q4V896 | Sorting nexin-15 | *Snx15* | 0.0045 | -0.6716 |
| Q4V8B2 | DCN1-like protein 3 | *Dcun1d3* | 0.0041 | 0.3383 |
| Q4V8E1 | GATA zinc finger domain-containing 2B | *Gatad2b* | 0.0061 | -0.3175 |
| Q4V8H8 | EH domain-containing protein 2 | *Ehd2* | 0.0057 | 0.3996 |
| Q56AP7 | Protein cereblon | *Crbn* | 0.0178 | 0.2709 |
| Q5EBC0 | Inter alpha-trypsin inhibitor, heavy chain 4 | *Itih4* | 0.0091 | 0.3128 |
| Q5EIC4 | Probable E3 ubiquitin-protein ligase IRF2BPL | *Irf2bpl* | 0.0421 | 0.2807 |
| Q5FWT5 | Glutamyl-tRNA(Gln) amidotransferase subunit A, mitochondrial | *Qrsl1* | 0.0490 | 0.3238 |
| Q5I0L3 | Tyrosine--tRNA ligase, mitochondrial | *Yars2* | 0.0002 | 0.5864 |
| Q5PPG2 | Legumain | *Lgmn* | 0.0067 | 0.3162 |
| Q5PPN5 | Tubulin polymerization-promoting protein family member 3 | *Tppp3* | 0.0444 | 0.3289 |
| Q5PQL2 | CCR4-NOT transcription complex subunit 9 | *Cnot9* | 0.0017 | -0.4059 |
| Q5PQS6 | Protein SMG9 | *Smg9* | 0.0333 | -0.2656 |
| Q5RK30 | Ribosome maturation protein SBDS | *Sbds* | 0.0062 | 0.3695 |
| Q5U2U4 | Secretory carrier-associated membrane protein | *Scamp2* | 0.0486 | -0.3034 |
| Q5U318 | Astrocytic phosphoprotein PEA-15 | *Pea15* | 0.0013 | 0.3600 |
| Q5XI20 | Maturin | *Mturn* | 0.0071 | 0.6230 |
| Q5XI29 | Cleavage and polyadenylation specificity factor subunit 7 | *Cpsf7* | 0.0088 | -0.4616 |
| Q5XIB1 | Protein TSSC4 | *Tssc4* | 0.0484 | -0.5884 |
| Q5XIE0 | Acidic leucine-rich nuclear phosphoprotein 32 family member E | *Anp32e* | 0.0089 | 0.2877 |
| Q5XIF4 | Small ubiquitin-related modifier 3 | *Sumo3* | 0.0030 | -0.2934 |
| Q5XIJ6 | BRISC and BRCA1-A complex member 1 | *Babam1* | 0.0219 | -0.3234 |
| Q62845 | Contactin-4 | *Cntn4* | 0.0001 | 0.2678 |
| Q63450 | Calcium/calmodulin-dependent protein kinase type 1 | *Camk1* | 0.0145 | 0.2775 |
| Q63487 | Ras-related GTP-binding protein B | *RragB* | 0.0069 | 0.3073 |
| Q63488 | Sodium-dependent phosphate transporter 2 | *Slc20a2* | 0.0248 | -0.3885 |
| Q63910 | Alpha globin | *Hba-a3* | 0.0093 | 0.5306 |
| Q63965 | Sideroflexin-1 | *Sfxn1* | 0.0133 | 0.4692 |
| Q642E6 | Tripeptidyl peptidase I | *Tpp1* | 0.0220 | -0.4021 |
| Q64350 | Translation initiation factor eIF-2B subunit epsilon | *Eif2b5* | 0.0026 | 0.6899 |
| Q66H41 | RCG28719 | *Snx7* | 0.0326 | -0.3080 |
| Q6AXS5 | Plasminogen activator inhibitor 1 RNA-binding protein | *Serbp1* | 0.0121 | -0.2634 |
| Q6AY19 | Atypical kinase COQ8B, mitochondrial | *Coq8b* | 0.0377 | 0.4093 |
| Q6AYD9 | Nucleoside diphosphate-linked moiety X motif 19 | *Nudt19* | 0.0205 | 0.3447 |
| Q6AYP7 | 7-methylguanosine phosphate-specific 5-nucleotidase | *Nt5c3b* | 0.0043 | 0.3126 |
| Q6JP77 | A-kinase anchor protein 7 isoforms delta and gamma | *Akap7* | 0.0424 | -0.2949 |
| Q6MG51 | Uncharacterized protein C6orf47 homolog | *G4* | 0.0019 | -0.2897 |
| Q6MG61 | Chloride intracellular channel protein 1 | *Clic1* | 0.0049 | 0.2710 |
| Q6P4Z9 | COP9 signalosome complex subunit 8 | *Cops8* | 0.0482 | 0.2670 |
| Q761X5 | Netrin receptor UNC5C | *Unc5c* | 0.0016 | -0.4061 |
| Q78PB6 | Nuclear distribution protein nudE-like 1 | *Ndel1* | 0.0028 | 0.2906 |
| Q812D3 | Peptidyl-prolyl cis-trans isomerase-like 3 | *Ppil3* | 0.0005 | 0.3053 |
| Q8CHN7 | Calmodulin regulator protein PCP4 | *Pcp4* | 0.0120 | -0.3790 |
| Q8R462 | Amino acid transporter (Fragment) | *0.0008* | -0.9519 |  |
| Q99PV2 | Syntaxin binding protein 3, isoform CRA_a | *Stxbp3* | 0.0066 | 0.4595 |
| Q9QZA6 | CD151 antigen | *Cd151* | 0.0298 | 0.3445 |
| Q9WTT7 | Basic leucine zipper and W2 domain-containing protein 2 | *Bzw2* | 0.0015 | 0.4368 |
| Q9WUC4 | Copper transport protein ATOX1 | *Atox1* | 0.0151 | -0.3156 |
| Q9Z0V5 | Peroxiredoxin-4 | *Prdx4* | 0.0385 | 0.3169 |
| **Morphine-DBS vs. Saline** |  |  |  |  |
| A0A0G2JV09 | Synembryn-B | *Ric8b* | 0.0203 | -0.2698 |
| A0A0G2JVK3 | 5-AMP-activated protein kinase subunit beta-2 | *Prkab2* | 0.0090 | 0.2946 |
| A0A0G2JWG6 | Golgin B1 | *Golgb1* | 0.0061 | -0.4557 |
| A0A0G2JYI0 | LPS-responsive beige-like anchor protein | *Lrba* | 0.0279 | -0.3019 |
| A0A0G2JZB7 | Neuron navigator 3 | *Nav3* | 0.0043 | -0.3572 |
| A0A0G2K121 | Unc-119 lipid-binding chaperone B | *Mlec* | 0.0038 | -0.2933 |
| A0A0G2K1L4 | RALBP1 associated Eps domain containing protein 2 | *Reps2* | 0.0372 | -0.2958 |
| A0A0G2K5N8 | ArfGAP with coiled-coil, ankyrin repeat and PH domains 3 | *Acap3* | 0.0036 | -0.2933 |
| A0A0G2K9B1 | Serine protease inhibitor A3N | *LOC299282* | 0.0316 | 0.3171 |
| A0A0G2QC15 | HIV-1 Tat interactive protein 2 | *Htatip2* | 0.0305 | 0.4183 |
| A0A0H2UHP1 | Retinal dehydrogenase 1 | *Aldh1a1* | 0.0262 | -0.4084 |
| A0A1W2Q678 | Uncharacterized protein | *FAM120C* | 0.0106 | 0.3320 |
| A1L1I6 | Reticulon | *Rtn3* | 0.0434 | 0.3443 |
| B0BMZ5 | LOC687696 protein | *Stambpl1* | 0.0284 | -0.3287 |
| B0BN15 | Similar to src homology 2 domain-containing transforming protein D | *Shd* | 0.0014 | -0.3864 |
| B0K025 | Oligosaccharyltransferase complex subunit OSTC | *Ostc* | 0.0106 | 0.4156 |
| B1WBM0 | Tetraspanin | *Cd9* | 0.0357 | 0.4262 |
| B2RYI8 | Papss1 protein | *Papss1* | 0.0064 | -0.2707 |
| B2RYT9 | Translational activator of cytochrome c oxidase 1 | *Taco1* | 0.0021 | 0.6602 |
| B2RYV2 | Nudt16 protein | *Nudt16* | 0.0418 | -0.3865 |
| B2RYW4 | Mitochondrial ribosomal protein L53 | *Mrpl53* | 0.0012 | -0.3980 |
| B2RZ61 | Carbonic anhydrase 7 | *Car7* | 0.0047 | -0.3436 |
| B5DEM5 | 60S ribosomal protein L14 | *Rpl14* | 0.0232 | -0.4015 |
| D1M8S3 | Interleukin 1 receptor accessory protein b | *Il1rap* | 0.0323 | -0.3268 |
| D3Z890 | Sn1-specific diacylglycerol lipase beta | *Daglb* | 0.0245 | -0.5280 |
| D3Z949 | Similar to RIKEN cDNA 2900010M23 | *RGD1561671* | 0.0343 | 0.3060 |
| D3ZB51 | Protein turtle homolog B | *Igsf9b* | 0.0254 | 0.3224 |
| D3ZBN0 | Histone H1.5 | *H1-5* | 0.0034 | -0.3115 |
| D3ZCL3 | U1 small nuclear ribonucleoprotein C | *Snrpc* | 0.0076 | -0.2987 |
| D3ZHA0 | Filamin-C | *Flnc* | 0.0088 | 0.3652 |
| D3ZHV7 | Serine dehydratase-like | *Sdsl* | 0.0125 | 0.3938 |
| D3ZIJ5 | Dolichol-phosphate mannosyltransferase subunit 3 | *Dpm3* | 0.0073 | -0.2848 |
| D3ZJ92 | Pre-mRNA processing factor 40 homolog A (Yeast) (Predicted) | *Prpf40a* | 0.0163 | -0.2884 |
| D3ZKT0 | Phosphatidate cytidylyltransferase, mitochondrial | *Tamm41* | 0.0012 | 0.2741 |
| D3ZWU9 | Required for meiotic nuclear division 5 homolog A | *Rmnd5a* | 0.0122 | 0.4222 |
| D3ZXJ5 | Elongation factor-like GTPase 1 | *Efl1* | 0.0053 | -0.2716 |
| D3ZYR1 | F-BAR domain only protein 2 | *Fcho2* | 0.0016 | -0.3542 |
| D4A2K5 | Nuclear receptor interacting protein 3 (Predicted), isoform CRA_a | *Nrip3* | 0.0001 | -0.3659 |
| D4A3X6 | Phospholipid-transporting ATPase | *Atp8a2* | 0.0076 | -0.2775 |
| D4A626 | Calmin | *Clmn* | 0.0026 | 0.3250 |
| D4A997 | HIV TAT specific factor 1 (Predicted) | *Htatsf1* | 0.0160 | -0.3188 |
| D4A9Q5 | Carboxypeptidase M | *Cpm* | 0.0001 | 0.6208 |
| D4ABB2 | Megalencephalic leukoencephalopathy with subcortical cysts 1 homolog (Human) (Predicted) | *Mlc1* | 0.0250 | 0.2636 |
| D4ABM5 | Mitochondrial ribosomal protein S34 | *Mrps34* | 0.0314 | -0.3145 |
| D4ABY0 | Complexin 3 | *Cplx3* | 0.0069 | -0.2894 |
| D4ADS4 | Microsomal glutathione S-transferase 3 | *Mgst3* | 0.0013 | 0.2849 |
| D4AEL3 | Distal membrane arm assembly complex 1 | *Dmac1* | 0.0032 | -0.5122 |
| E2CWF0 | Serine/threonine-protein phosphatase | *Ppp3cc* | 0.0320 | -0.2689 |
| F1LQS3 | 60S ribosomal protein L6 | *Rpl6-ps1* | 0.0224 | -0.3537 |
| F1M0N1 | Tyrosine-protein kinase | *Abl2* | 0.0178 | -0.3262 |
| F1M1B3 | WASH complex subunit 5 | *Washc5* | 0.0059 | 0.2755 |
| F1M1W4 | Nedd4 family-interacting protein 2 | *Ndfip2* | 0.0333 | -0.2999 |
| F1M3B1 | Ankyrin repeat domain-containing protein 29-like | *LOC108348154* | 0.0397 | -0.2929 |
| F1M3H8 | Hypothetical protein LOC681410 | *LOC681410* | 0.0028 | 0.4960 |
| F1M6T3 | Junctional cadherin 5-associated | *Jcad* | 0.0055 | -0.3833 |
| F2W8A6 | Neuropeptide Y | *Npy* | 0.0029 | 0.7119 |
| F6Q5K7 | Mitochondrial ribosomal protein S18B | *Mrps18b* | 0.0317 | -0.2965 |
| G3V6L5 | Protein tyrosine phosphatase, receptor type, R, isoform CRA_d | *Ptprr* | 0.0431 | -0.3497 |
| G3V6Y6 | Alpha-1,4 glucan phosphorylase | *Pygb* | 0.0461 | 0.3396 |
| G3V6Z5 | Glutamate receptor 3 | *Gria3* | 0.0002 | -0.2712 |
| G3V790 | Transcription activator BRG1 | *Smarca4* | 0.0065 | -0.3255 |
| G3V7W1 | Programmed cell death protein 6 | *Pdcd6* | 0.0002 | 0.2907 |
| G3V8K2 | Guanine nucleotide-binding protein subunit gamma | *Gng3* | 0.0163 | 0.2711 |
| G3V913 | Heat shock 27kDa protein 1 | *Hspb1* | 0.0005 | 0.8044 |
| G3V940 | Coronin | *Coro1b* | 0.0017 | 0.3317 |
| K7S2S2 | Histone H2A | *Hist2h2aa3* | 0.0486 | 0.2850 |
| M0R665 | 60S ribosomal protein L29 | *0.0152* | -0.2662 |  |
| M0R835 | Splicing factor 3B, subunit 6 | *Sf3b6* | 0.0314 | -0.4152 |
| M0RA99 | Receptor-type tyrosine-protein phosphatase S | *Ptprs* | 0.0434 | -0.3005 |
| M0RAD5 | ATP-dependent Clp protease proteolytic subunit | *Clpp* | 0.0155 | 0.2885 |
| M0RAT6 | Transmembrane p24 trafficking protein 8 | *Tmed8* | 0.0232 | 0.3619 |
| O35263 | Platelet-activating factor acetylhydrolase IB subunit gamma | *Pafah1b3* | 0.0211 | 0.2711 |
| O70352 | CD82 antigen | *Cd82* | 0.0046 | 0.3252 |
| O88279 | Slit homolog 1 protein | *Slit1* | 0.0194 | 0.3678 |
| O88658 | Kinesin-like protein KIF1B | *Kif1b* | 0.0049 | 0.3207 |
| O88794 | Pyridoxine-5-phosphate oxidase | *Pnpo* | 0.0385 | -0.2710 |
| P01048 | T-kininogen 1 | *Map1* | 0.0048 | 0.7053 |
| P02625 | Parvalbumin alpha | *Pvalb* | 0.0363 | -0.5830 |
| P06536 | Glucocorticoid receptor | *Nr3c1* | 0.0140 | 0.4205 |
| P07151 | Beta-2-microglobulin | *B2m* | 0.0308 | 0.3111 |
| P07171 | Calbindin | *Calb1* | 0.0017 | -0.3309 |
| P08483 | Muscarinic acetylcholine receptor M3 | *Chrm3* | 0.0394 | -0.3016 |
| P0C0A9 | Small VCP/p97-interacting protein | *Svip* | 0.0124 | 0.2665 |
| P11348 | Dihydropteridine reductase | *Qdpr* | 0.0180 | 0.2777 |
| P20059 | Hemopexin | *Hpx* | 0.0244 | 0.4094 |
| P47196 | RAC-alpha serine/threonine-protein kinase | *Akt1* | 0.0128 | 0.3579 |
| P47819 | Glial fibrillary acidic protein | *Gfap* | 0.0202 | 0.4916 |
| P60756 | MAM domain-containing glycosylphosphatidylinositol anchor protein 2 | *Mdga2* | 0.0275 | -0.3506 |
| P62329 | Thymosin beta-4 | *Tmsb4x* | 0.0459 | 0.2860 |
| P62775 | Myotrophin | *Mtpn* | 0.0131 | -0.2808 |
| P80299 | Bifunctional epoxide hydrolase 2 | *Ephx2* | 0.0071 | -0.4206 |
| P80432 | Cytochrome c oxidase subunit 7C, mitochondrial | *Cox7c* | 0.0029 | -0.3029 |
| Q02765 | Cathepsin S | *Ctss* | 0.0238 | 0.3190 |
| Q1RP74 | RCG53953, isoform CRA_a | *LOC103690005* | 0.0230 | -0.3338 |
| Q32PX9 | AFG1-like ATPase | *Afg1l* | 0.0166 | 0.2670 |
| Q3B8Q1 | Nucleolar RNA helicase 2 | *Ddx21* | 0.0060 | -0.3101 |
| Q3KRE2 | Methyltransferase like 7A, isoform CRA_b | *Mettl7a* | 0.0305 | 0.5543 |
| Q3KRE8 | Tubulin beta-2B chain | *Tubb2b* | 0.0042 | -0.2999 |
| Q3ZAU5 | DDHD domain-containing 1 | *Ddhd1* | 0.0212 | -0.3183 |
| Q4KM45 | UPF0687 protein C20orf27 homolog | *0.0326* | 0.2852 |  |
| Q4V896 | Sorting nexin-15 | *Snx15* | 0.0196 | -0.8760 |
| Q4V8B2 | DCN1-like protein 3 | *Dcun1d3* | 0.0169 | 0.3048 |
| Q4V8E1 | GATA zinc finger domain-containing 2B | *Gatad2b* | 0.0366 | -0.3130 |
| Q4V8H7 | E3 ubiquitin-protein ligase | *Wwp1* | 0.0187 | -0.2995 |
| Q56A26 | Oligodendrocytic myelin paranodal and inner loop protein | *Opalin* | 0.0017 | 0.4708 |
| Q5BJM5 | Pleckstrin homology domain-containing family O member 1 | *Plekho1* | 0.0394 | -0.3803 |
| Q5I0L3 | Tyrosine--tRNA ligase, mitochondrial | *Yars2* | 0.0026 | 0.3997 |
| Q5PQN7 | Protein LZIC | *Lzic* | 0.0412 | -0.2759 |
| Q5RK30 | Ribosome maturation protein SBDS | *Sbds* | 0.0064 | 0.3298 |
| Q5U2U4 | Secretory carrier-associated membrane protein | *Scamp2* | 0.0253 | -0.5210 |
| Q5U318 | Astrocytic phosphoprotein PEA-15 | *Pea15* | 0.0093 | 0.2823 |
| Q5XI20 | Maturin | *Mturn* | 0.0065 | 0.5714 |
| Q5XI29 | Cleavage and polyadenylation specificity factor subunit 7 | *Cpsf7* | 0.0158 | -0.4710 |
| Q5XIC2 | Evolutionarily conserved signaling intermediate in Toll pathway, mitochondrial | *Ecsit* | 0.0443 | -0.3749 |
| Q5XIE3 | 39S ribosomal protein L11, mitochondrial | *Mrpl11* | 0.0018 | -0.3740 |
| Q5XIJ6 | BRISC and BRCA1-A complex member 1 | *Babam1* | 0.0260 | -0.3029 |
| Q62706 | Nonmuscle myosin heavy chain-B (Fragment) | *0.0375* | -0.4409 |  |
| Q63226 | Glutamate receptor ionotropic, delta-2 | *Grid2* | 0.0063 | -0.4113 |
| Q63487 | Ras-related GTP-binding protein B | *RragB* | 0.0037 | 0.3140 |
| Q642E6 | Tripeptidyl peptidase I | *Tpp1* | 0.0026 | -0.4642 |
| Q64680 | Cytochrome P450 2D4 | *Cyp2d4* | 0.0342 | -0.3055 |
| Q6JP77 | A-kinase anchor protein 7 isoforms delta and gamma | *Akap7* | 0.0437 | -0.2658 |
| Q6MG61 | Chloride intracellular channel protein 1 | *Clic1* | 0.0077 | 0.2656 |
| Q7TP06 | Da1-6 |  | 0.0087 | 0.2894 |
| Q8CHN7 | Calmodulin regulator protein PCP4 | *Pcp4* | 0.0403 | -0.3112 |
| Q8R462 | Amino acid transporter (Fragment) | *0.0338* | -0.5568 |  |
| Q99PV2 | Syntaxin binding protein 3, isoform CRA_a | *Stxbp3* | 0.0097 | 0.4162 |
| Q9JJK4 | Peroxisomal biogenesis factor 3 | *Pex3* | 0.0063 | 0.4152 |
| Q9QZA6 | CD151 antigen | *Cd151* | 0.0014 | 0.3992 |
| Q9WUC4 | Copper transport protein ATOX1 | *Atox1* | 0.0052 | -0.3805 |
